# The Role of Personalised Choice in Decision Support: A Randomized Controlled Trial of an Online Decision Aid for Prostate Cancer Screening

**DOI:** 10.1371/journal.pone.0152999

**Published:** 2016-04-06

**Authors:** Glenn Salkeld, Michelle Cunich, Jack Dowie, Kirsten Howard, Manish I. Patel, Graham Mann, Wendy Lipworth

**Affiliations:** 1 Faculty of Social Sciences, University Of Wollongong, Wollongong, NSW, Australia; 2 Faculty of Pharmacy and Charles Perkins Centre, University of Sydney, Sydney, NSW, Australia; 3 London School of Hygiene and Tropical Medicine, London, United Kingdom; 4 Sydney School of Public Health, University of Sydney, Sydney, NSW, Australia; 5 Westmead Clinical School, Westmead Hospital, Sydney, NSW, Australia; 6 Westmead Institute for Medical Research, Westmead Hospital, Sydney, NSW, Australia; 7 Centre for Values, Ethics and the Law in Medicine, University of Sydney, Sydney, NSW, Australia; Innsbruck Medical University, AUSTRIA

## Abstract

**Importance:**

Decision support tools can assist people to apply population-based evidence on benefits and harms to individual health decisions. A key question is whether “personalising” choice within decisions aids leads to better decision quality.

**Objective:**

To assess the effect of personalising the content of a decision aid for prostate cancer screening using the Prostate Specific Antigen (PSA) test.

**Design:**

Randomized controlled trial.

**Setting:**

Australia.

**Participants:**

1,970 men aged 40–69 years were approached to participate in the trial.

**Intervention:**

1,447 men were randomly allocated to either a standard decision aid with a fixed set of five attributes or a personalised decision aid with choice over the inclusion of up to 10 attributes.

**Outcome Measures:**

To determine whether there was a difference between the two groups in terms of: 1) the emergent opinion (generated by the decision aid) to have a PSA test or not; 2) self-rated decision quality after completing the online decision aid; 3) their intention to undergo screening in the next 12 months. We also wanted to determine whether men in the personalised choice group made use of the extra decision attributes.

**Results:**

5% of men in the fixed attribute group scored ‘Have a PSA test’ as the opinion generated by the aid, as compared to 62% of men in the personalised choice group (χ^2^ = 569.38, 2df, p< 0001). Those men who used the personalised decision aid had slightly higher decision quality (t = 2.157, df = 1444, p = 0.031). The men in the personalised choice group made extensive use of the additional decision attributes. There was no difference between the two groups in terms of their stated intention to undergo screening in the next 12 months.

**Conclusions:**

Together, these findings suggest that personalised decision support systems could be an important development in shared decision-making and patient-centered care.

**Trial Registration:**

Australian New Zealand Clinical Trials Registry (ANZCTR) ACTRN12612000723886

## Background

### Clinical decision support for cancer screening tests

In an era of “shared decision-making” and “patient centered care,” it is widely accepted that citizens have the right to make informed choices about all aspects of health care, including whether or not to undergo screening tests [1, 2]. Information relevant to screening decisions can, however, be complex and, as Entwistle *et al* [3] and others [4, 5] have noted, not everyone either wants to or is capable of analyzing research data on the pros and cons of screening, process numeric information on risk, deal with uncertainty relating to potential benefits and harms, and then make an “informed” choice about screening. Paper-based, video and on-line decision support tools can assist members of the public to interpret numeric information (probability) on the risks and benefits of screening and deal with uncertainty. They do this by providing a framework for analysis that reduces some of the cognitive demands of processing information on probability [6].

### The quality of decision aids

Not all decision aids are equally effective in assisting decision-making, and it is important that their quality is assessed prior to implementation[7]. “Decision attributes” that might be used to assess the quality of a decision aid include measures of knowledge and risk perception; whether participants feel informed and clear about values; whether they feel certain about and satisfied with a decision; whether they subsequently participate actively in decision-making; and whether clinician—patient communication is improved. Relevant outcome measures include persistence with chosen therapy, quality of life, and healthcare costs [6].

The quality of decision aids can be enhanced, for example, by ensuring that they are sufficiently detailed and that they contain explicit values clarification exercises [6]. There are, however, still many unanswered questions about the best way to design decision aids [6]. One specific question about decision aids that remains unanswered is whether there is any benefit in “personalising” choice within decision aids. That is, whether it is beneficial to allow participants to choose which factors are important to them in making decisions, and therefore which attributes should be included in decision aids. The premise underpinning this approach, and the starting point for shared decision-making and patient centered care, is that it is important to focus on what individuals know best—their own values—and then to help them develop their ability to express personal preferences in the context of making an informed health decision [8].

In this paper we report the results of a randomized controlled trial designed to assess the effects of personalising the content of an online interactive decision aid for prostate cancer screening using the Prostate Specific Antigen test (PSA). Specifically, we were interested in exploring the effects of personalization on 1) the emergent, decision aid-generated, opinion as to whether or not to pursue PSA testing, 2) the self-rated quality of the decision made, 3) the process of reasoning about PSA testing that led to the particular opinion, and 4) the intention to undergo screening in the next 12 months.

### Prostate cancer screening and decision support

Many clinicians believe that prostate cancer is best detected as early as possible through screening of aymptomatic men, using prostate specific antigen (PSA) testing and/or digital rectal examination (DRE). Although PSA is a non-specific biomarker, which can also be abnormal as a result of other conditions such as infection, PSA screening (and/or DRE) are currently the only methods available for screening for prostate cancer in asymptomatic men and routine screening using PSA remains common. (While there are other tests and prediction tools for prostate cancer, these serve different purposes. For example, Partin tables predict the final pathology following prostatectomy based on preoperative variables. European Organization for Research and Treatment of Cancer (EORTC) risk tables predict the likelihood of having cancer in a biopsy based on PSA and other variables. Other factors, such as free PSA (fPSA) and prostate volume, are also used subsequent to screening (using PSA or DRE) to predict the likelihood of prostate cancer. None of these tests are *screening* tests. Other potential *screening* tools include PSA isoforms, Prostate Cancer Antigen 3 (PCA3 or DD3) and magnetic resonance imaging, however these have not yet been comprehensively evaluated and are not used routinely for screening).

Despite the frequency with which PSA screening is conducted, evidence has emerged over the past few years that questions the appropriateness of routine PSA screening for prostate cancer [9–11]. While some research suggests significant mortality reductions associated with PSA screening, other research suggests there is no mortality reduction associated with screening or at least not one that justifies the risks associated with testing and treatment. For example, Andriole et al (2012), in an analysis of the 13 year follow-up of the Prostate, Lung, Colorectal, and Ovarian (PLCO) trial, found no evidence of a mortality benefit for organized annual screening compared to opportunistic screening (as part of usual care) [12]. In 2013, the authors of a Cochrane Review concluded that PSA screening does not significantly reduce mortality, and is often harmful [13].

Despite this, there is evidence that routine PSA testing remains common in some populations. Williams et al (2010), for example, report PSA testing rates in UK general practices to be 1.4% (95% CI 1.1–1.6%) in men aged 45–49 years, but rising sharply to 11.3% (95% CI 10.0–12.9%) in men aged 75–79 years (p-value for trend <0.001) [14]. A recent US study showed that more than 50% of healthy men aged 65–74 years were screened in 2010 [15]. An Australian study of men under 55 years found that PSA testing increased by 146% between 2001 and 2008 [11].

In an effort to bridge the gap between evidence and practice, most major policymaking organisations internationally now recommend against routine prostate cancer screening using PSA [16–23]. If a man asks his doctor to be tested, then it is recommended that testing take place only after a thorough process of shared decision-making and informed consent. Urological associations internationally often take a less definite position on screening, but they too tend to recommend against general population screening programs, and emphasise the need for shared decision making for men who are in high risk groups or who ask to be tested [24, 25].

A number of decision support tools have been developed to facilitate this kind of shared decision-making for prostate cancer screening. These include written, online and video materials, which are sometimes combined with education and discussion sessions. Decision aids usually present information about the risks of prostate cancer, and the risks and benefits of various relevant tests and treatments. The decision attributes that are most commonly included are the perceived importance (or lack thereof) of: extending life; avoiding death from prostate cancer; early diagnosis and treatment; avoiding urinary, bowel or sexual dysfunction stemming from diagnosis and treatment; avoiding false positives resulting in unnecessary anxiety, tests (e.g. biopsies) and treatments; avoiding false negatives and false reassurance (sometimes framed as the importance of having an accurate screening test); being active in caring for one’s health; having knowledge; having peace of mind; and being “safe” rather than “sorry” ^e.g.^ [26–35].

Numerous trials have been conducted into the effects of providing decision support for PSA testing. These trials have generally found that men who use decision support tools are better informed about prostate cancer and screening, more confident about their decision, less conflicted in their decision-making, and more likely to participate actively in decision-making. They are also less likely to express an intention to be screened and, subsequently, less likely to opt for PSA testing as part of routine care, although this is somewhat variable (e.g. it is less likely to be the case for men who are specifically seeking screening services) [6, 26, 36–43]. Although there are numerous existing decision support tools for prostate cancer, which have been compared to standard care and to each other, these tools all have in common that the decision attributes are pre-defined and people using the tools have no choice but to consider all attributes that are presented to them. In other words, they are not “personalised” in the sense described previously. We therefore chose to develop and test the effects of a personalised decision aid for PSA screening.

## Method

### Phase 1: Developing a personalised decision aid for prostate cancer screening

#### The Annalisa decision support platform

The personalised decision support tool that we developed for prostate cancer screening used a software platform known as Annalisa—an interactive decision aid template based on Multi Criteria Decision Analysis (MCDA) [44, 45]. This approach was developed in collaboration with clinicians and patients [44], and recognizes that there are often multiple and competing criteria that drive decision-making. Methods applied within the MCDA framework assess the individual’s “trade offs” among these criteria and then generate an “opinion” based on the trade offs that are made [46–48].

**Fig 1:**
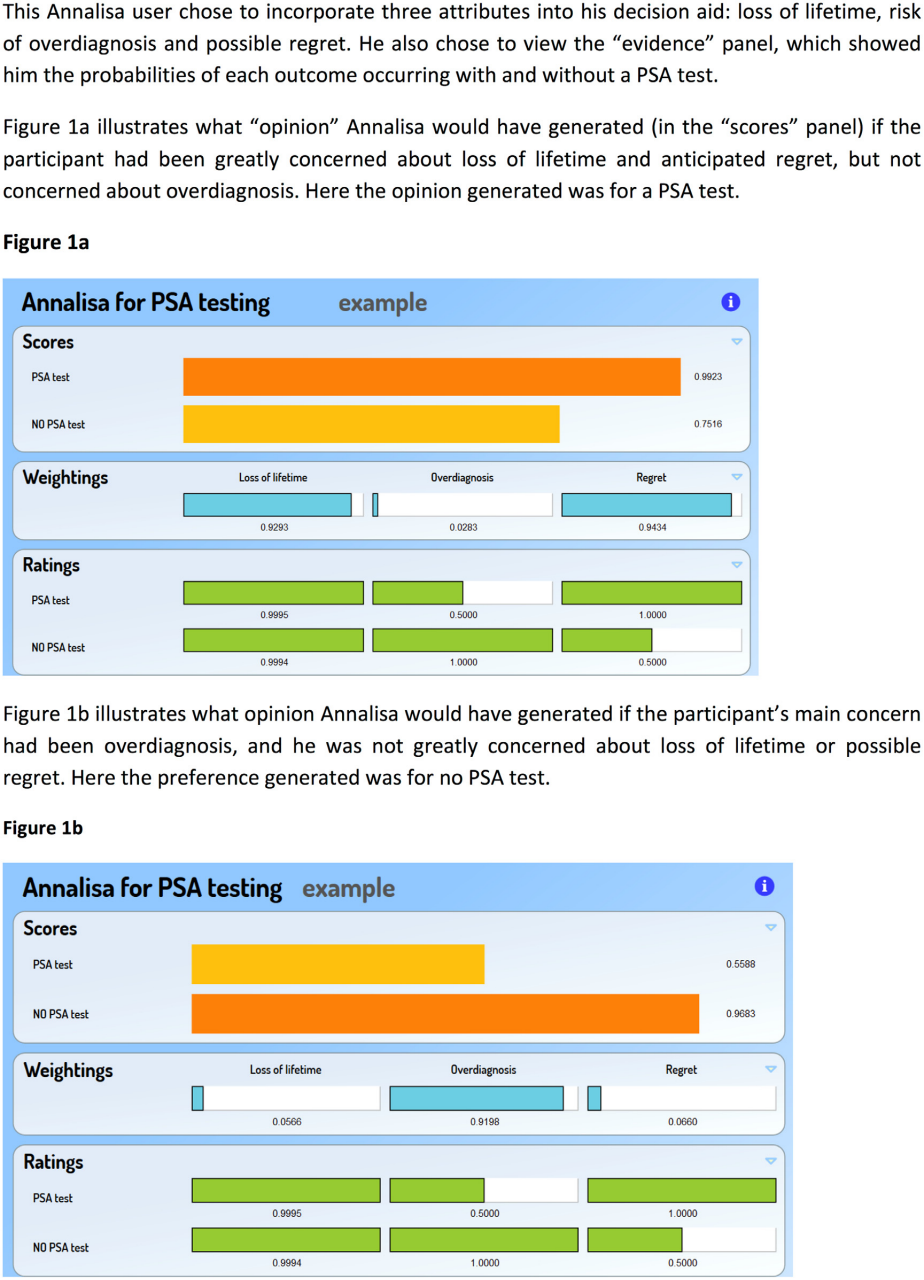
Demonstration of Annalisa decision tool for Prostate Cancer Screening.

Annalisa, an example of a prescriptive decision aid, presents users with a set of relevant decision attributes. They are then asked to indicate the relative importance or weight they wish to attach to the attribute by dragging the cursor associated with each attribute to the left (lower weight) or to the right (higher weight) on a “Weightings Panel.” If they want to see it, the participant is also given pre-populated information (expressed as probabilities of particular outcomes) about each attribute on a “Ratings Panel.” A bar graph is then generated on a “Scores Panel” that shows the emergent opinion based on a summed multiplication for each option of evidence ratings and the user’s weightings assigned to each attribute **(**Fig 1). An overall opinion is then generated by the software, which is rated and ranked compared to other options. In this study, the opinions generated were whether or not to undergo PSA testing. A more detailed description and demonstration of Annalisa can be found at: **healthedecisions.org.au**.

Annalisa differs from standard decision aids (both descriptive and prescriptive) in that it does not assume that participants know and understand risk (that is, probabilities that express the chance of an event occurring). It is well recognized that statistical illiteracy and “probability blindness” are major challenges in making informed health choices, and that these are not easily remedied through more education and more transparent representation of chance [4]. Rather than trying to simultaneously achieve statistical literacy and preference elicitation, therefore, the Annalisa decision aid simplifies decision support to just one task—asking the participant to express their importance weight for each attribute (benefit or harm) related to the options. Information on the best available evidence for screening, expressed as the probability of an event or outcome occurring, can be viewed online. However, the user does not need to view this information in order to generate the score for each option. An algorithm embedded in the decision aid automatically carries out the simple “expected value” calculation, which involves multiplying the probability of the event occurring by the importance weight.

### Phase 2: Randomized trial of the personalised decision aid

A pilot study was conducted to ensure that the tool was user-friendly and that men understood the meaning of the 5 fixed and 5 additional attributes. Once the pilot study had been completed, 1,970 men were invited to participate in the study. Each participant was directed to an online survey and asked to give consent to participate in the study. Upon consenting to participate, men who were eligible were block randomized by age group (40-49yrs, 50-59yrs or 60-69yrs) to either: 1) the fixed attribute group (active comparator) or 2) the personalised choice group (active intervention). Computer randomization was undertaken to enroll participants in either the active comparator or active intervention group.

### Online survey

An online survey was developed (with the PSA decision aid embedded within it) in order to screen out candidates who did not meet the eligibility criteria and stratify people according to age and risk of prostate cancer based on family history. The survey was identical for both trial groups (S6 File). Participants were asked whether they had ever had a PSA test and when; the likelihood they would consult a GP within the next 12 months about having a PSA test; as well as information on their socio demographic characteristics and personal and family history of prostate cancer, which were important for determining eligibility and probabilities of risk and benefit.

### Randomised trial

**Fig 2:**
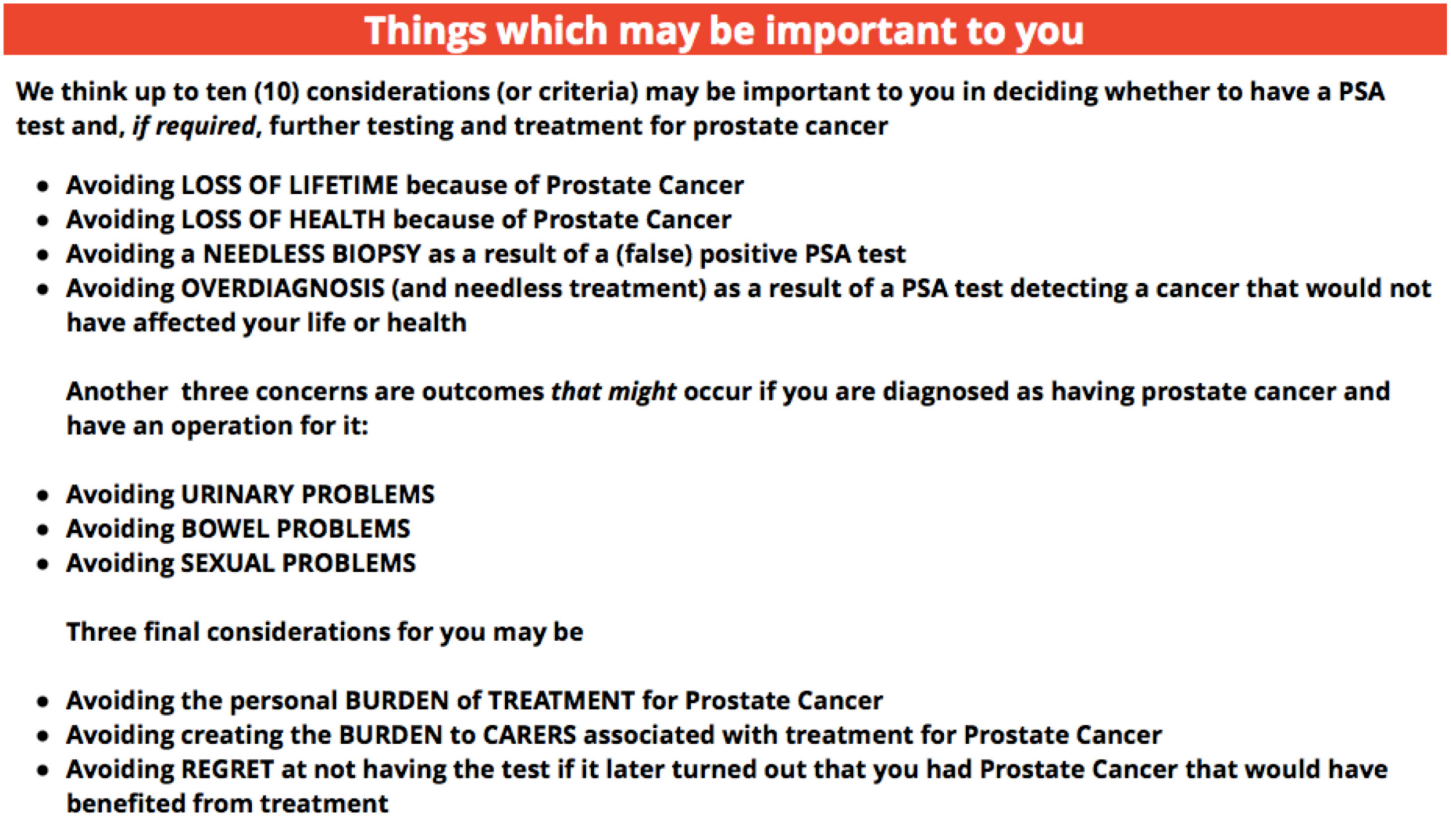
Description of attributes for the personalised decision aid.

Two version of Annalisa were developed: (1) a fixed attributes version consisting of the five fixed attributes: survival (lifetime), needless biopsy, and urinary, bowel and sexual functioning problems, and (2) a personalised choice version consisting of the 5 fixed attributes plus 5 extra attributes: quality of life, overdiagnosis, burden of treatment, burden to carers, and anticipated regret. The process we used for personalising Annalisa is described in an E appendix (S10 File) and the attributes as they were presented to the participants are illustrated in Fig 2.

Those in the fixed attribute group were directed immediately to the Annalisa decision aid with its five pre-determined attributes, while those in the personalised choice group were first asked to select as few or as many attributes as they wanted to from the 10 options described above.

The ratings (probabilities) for each included attribute were personalised to each participant based on their response to a survey question on the number of first-degree (male) relatives who have been affected by prostate cancer, and their stated age group.

Trial participants in both groups were then asked to weight the importance of each of the included attributes on a continuous scale from zero (no importance) to one (all important) in the context of a hypothetical decision on whether to have a PSA test.

#### Outcome measures

The two main outcome measures of the study were the option ranking (emergent opinion to have a PSA test or not have a PSA test as determined by the Annalisa tool) and (self-rated) decision quality after completing the online decision aid for PSA screening. The decision quality instrument MyDecisionQuality (MDQ)[49] was used to assess decision quality. MDQ is a generic (as opposed to condition-specific) tool for assessing decisions made using any form of decision technology. Like the PSA decision aid itself, MDQ uses the principles of Multi-Criterion Decision Analysis (MCDA) to weight and rate eight elements of decision quality, namely: perceived clarity about the nature of the available options, their effects, their personal importance and their likelihood; perceived ability to trust the information provided; perceived adequacy of support provided during decision making; perceived sense of control over the decision; and perceived degree of commitment to acting on the decision [49]. The participant was responsible for both weighting the criteria of decision quality in terms of their relative importance to them (prior to using the decision aid), and rating the quality of the decision just made according to these criteria (after completing the decision aid). A full description of each decision quality attribute can be found in the online supplementary material (S7 and S8 Files).

We were also interested in whether, and to what extent, men in the personalised choice group made use of the extra decision attributes in addition to, or instead of, the five fixed attributes and whether there was a difference between the two groups in terms of their intention to undergo PSA screening (irrespective of whatever opinion had been generated by the aid).

#### Recruitment, sample size and study participants

A sample size of 699 in each of the two arms of the trial was based on the number needed to detect a 0.15 difference in the mean Decision Quality (DQ) score that rejects the null hypothesis that the mean DQ scores are equal, with probability (power) 0.8. The potential study population consisted of 130,000 people from a market research company panel of Australians. To allow for non-consenters and men with prostate cancer diagnoses and hence ineligible for PSA screening, 1,970 men were contacted, of whom 1,447 eligible men consented to participate (73.5% response rate). Recruitment began on June 3, 2011 and concluded on June 21,2011.

#### Statistical analysis

Univariate analysis was performed. Differences in PSA test opinions (generated by the decision aid) and behavioral intentions between the two groups were compared using chi-square tests (p-values reported). Differences in the mean Decision Quality scores between the two groups was tested using a t-test. The mean (SD) importance weights (normalized) for each attribute in the decision aid were calculated and between group differences compared using a t-test.

The trial was approved by the University of Sydney Human Research Ethics Committee (Protocol No.: 05–2011 / 13712) on 13 May 2011 and was included in the Australian New Zealand Clinical Trials Registry (ANZCTR) on 6 July 2012 (ACTRN: ACTRN12612000723886). The authors confirm that all ongoing and related trials for this drug/intervention are registered. All participants gave informed consent.

## Results

A total of 1,970 men were invited to participate in the study. Of these, 1,010 were randomly allocated to the fixed attribute group and 960 to the personalised choice group. A total of 767 and 744 men respectively consented to participate in the study. There were 40 men in the fixed attribute group and 24 in the personalised choice group who were subsequently excluded because they had prostate cancer or were unsure whether they had prostate cancer or not. The final sample consisted of 727 men in the fixed attribute group and 720 men in the personalised choice group. A summary of respondent characteristics and socio demographics is provided in Table 1.

**Table 1:** Respondent Characteristics.

	All		Fixed Attributes		Personalised Choice	
	n = 1447		n = 727 (50.2%)		n = 720 (49.8%)	
	n	%	n	%	n	%
**Age**						
40–49 years	591	40.8	282	38.8	309	42.9
50–59 years	487	33.7	248	34.1	239	33.2
60–69 years	369	25.5	197	27.1	172	23.9
**Family history**						
None	1118	77.3	552	75.9	566	78.6
1	194	13.4	109	15.0	85	11.8
2 or more	14	1.0	5	0.7	9	1.3
Don't know	121	8.4	61	8.4	60	8.3
**Previous PSA testing**						
Had a PSA test in past 12 months	393	27.2	209	28.8	184	25.6
Had a PSA test longer than 12 months age	205	14.2	101	13.9	104	14.4
Never had a PSA test	768	53.1	382	52.5	386	53.6
Don’t Know	81	5.6	35	4.8	46	6.4
**General Health**						
Excellent	149	10.3	75	10.3	74	10.3
Very good	402	27.8	198	27.2	204	28.4
Good	587	40.6	302	41.5	285	39.7
Fair	232	16.1	118	16.2	114	15.9
Poor	75	5.2	34	4.7	41	5.7
**Time since last visited doctor (GP)**						
Less than 2 weeks	363	25.1	173	23.8	190	26.4
2 weeks to 3 months ago	492	34.0	253	34.8	239	33.2
3 to 6 months ago	257	17.8	145	19.9	112	15.6
6 to 12 months ago	165	11.4	81	11.1	84	11.5
12 months or more	150	10.4	67	9.2	83	11.5
Never	8	0.6	4	0.6	4	0.6
Not sure	11	0.8	4	0.6	4	0.6
**Health Cover (excluding Medicare)**						
Private health insurance with extras	551	38.8	279	39.1	272	38.6
Private health insurance without extras	94	6.6	45	6.3	49	7.0
Department of Veteran's Affairs card	36	2.5	14	2.0	22	3.12
Health care concession card	382	26.9	201	28.2	181	25.7
None of these	342	24.1	173	24.3	169	24.0
Don't know	14	0.99	2	0.3	12	1.7
**Country of Birth**						
Australia	1091	75.4	541	74.4	550	76.4
New Zealand	49	3.4	25	3.4	24	3.3
UK or Ireland	145	10.0	75	10.3	70	9.7
Elsewhere in Europe	71	4.9	42	5.8	29	4.0
Elsewhere in World	91	6.3	44	6.1	47	6.5
**Relationship Status**						
Married	768	53.1	393	54.1	375	52.1
De facto	149	10.3	83	11.4	66	9.2
Separated	70	4.9	23	3.2	47	6.3
Divorced	181	12.5	87	12.0	94	13.1
Widowed	25	1.7	14	1.9	11	1.5
Never married	254	17.6	127	17.5	127	17.6
**Highest Qualification**						
Higher deg./Postgrad diploma/Bachelors deg.	345	23.8	173	23.8	172	23.9
Undergraduate diploma/Associate diploma	262	18.1	128	17.6	134	18.6
Skilled/Basic vocational qualification	480	33.2	255	35.1	225	31.3
Has qualification but unsure about the level	71	4.9	32	4.4	39	5.4
No higher qualifications	289	20.0	139	19.1	150	20.8
**Employment Status**						
Not in the labour force	445	30.6	231	31.8	214	29.7
Employed	835	57.5	418	57.5	417	57.9
Unemployed	155	10.7	70	9.6	85	11.8
Don't know	12	0.8	8	1.1	4	0.6

### Survey results

There were no statistically significant differences between the two study groups in personal and other socio demographic characteristics. The average time taken to complete the survey online was 18.04 minutes for the fixed attribute group and 21:57 minutes for the personalised choice group, reflecting the additional time required to consider, select and rate the importance of the longer list of attributes in this arm.

### Decision aid results

The two main outcome measures for the trial were (1) the screening opinion as generated by the decision aid, and (2) the quality of the decision made using the aid (DQ) as measured on the MDQ (MyDecisionQuality) continuous scale.

#### Effects on the PSA screening opinion

There was a very clear difference in PSA screening opinion between the two groups. Only 4.7% of those in the fixed attribute (active comparator) group had an opinion generated that was in favor of the PSA test, compared to 61.5% in the personalised choice (active intervention) group (χ^2^ = 569.38, *2df*, *p< 0001)*. The difference in this screening opinion was clearly driven by the inclusion of attributes that are typically not measured in large randomized trials of cancer screening and testing, including overdiagnosis, burden of treatment, burden to carers, and anticipated regret (Table 2).

**Table 2:** Importance Weights (normalized) for each Attribute.

	Fixed Attributes				Personalised Choice					
	n = 727 (50.2%)				n = 720 (49.8%)					
	n	%	Mean	(SD)	n	%	Mean	(SD)	t stat (df)	p-value
**Avoiding (0–1):**										
Loss of Lifetime	726	100	0.354	(0.257)	635	88.2	0.148	(0.094)	-19.16 (df 1359)	<0.0001
Needless Biopsy	726	100	0.137	(0.121)	561	77.9	0.108	(0.062)	-5.18 (df 1285)	<0.0001
Bowel problems	726	100	0.168	(0.112)	661	91.8	0.121	(0.044)	-10.08 (df 1385)	<0.0001
Sexual problems	726	100	0.178	(0.154)	582	80.8	0.119	(0.062)	-8.66 (df 1306)	<0.0001
Urinary problems	726	100	0.163	(0.103)	650	90.3	0.118	(0.045)	-10.25 (df 1374)	<0.0001
Loss of health	n.a.				658	91.4	0.122	(0.055)		
Overdiagnosis	n.a.				556	77.2	0.101	(0.052)		
Regret	n.a.				565	78.5	0.114	(0.045)		
Treatment burden	n.a.				554	76.9	0.101	(0.041)		
Carer burden	n.a.				554	76.9	0.109	(0.055)		

#### Effects on decision quality

Respondent-rated decision quality was statistically significantly lower for the fixed attribute group at 0.67 than for the personalised choice group at 0.69 (t = 2.157,df = 1444, p = 0.031). Thus we reject the null hypothesis that the mean Decision Quality score is equal in both groups with probability (power) 0.8.

#### Other outcome measures

**Number of attributes selected:** Amongst the 720 respondents in the personalised choice group, 342 (47.5%) included all ten attributes in the decision aid, another 92 (12.8%) included nine attributes, 83 (11.5%) included eight attributes, 77 (10.7%) included seven attributes, 58 (8%) included six attributes, and the remaining 68 (9.5%) included five or fewer attributes in the decision aid.

**Reasons underpinning the opinion generated for or against PSA screening:** In both groups, survival (avoiding the loss of lifetime due to prostate cancer) received the highest importance rating of all attributes. In the fixed attribute group, survival achieved a weighting of 0.35. This decreased to just 0.15 in the personalised choice group when other attributes were also considered. The importance weights attached to these extra attributes were similar across the board (Table 2).

**Intention to have screening before and after using the decision aid:** When asked at the beginning of the survey (and before using the online decision aid) about the likelihood of having a PSA test in the next 12 months, both groups reported similar levels of intention (60.8% of men in the fixed attribute group and 58.1% of men in the personalised choice group stated they were likely or very likely to have a PSA test in the next 12 months). Despite the fact that only 4.7% of those in the fixed attribute (active comparator) group subsequently had an opinion generated from the aid in favor of PSA testing, almost as many men in the fixed attribute group (59.5%) as in the personalised choice group (68.3%) stated they were likely or very likely to have a PSA test in the next 12 months (Table 3). This suggests that even when men use decision aids that produce an opinion against PSA testing, they are likely to opt for testing when asked directly about their intentions.

**Table 3:** PSA test preferences and intentions.

	Fixed Attributes		Personalised Choice			
	n = 727 (50.2%)		n = 720 (49.8%)			
	n	%	n	%	Chi-square test stat (df)	p-value
**Highest scoring option**						
Have a PSA test	34	4.7	443	61.5	569.38 (df 2)	p<0.0001
Do not have a PSA test	692	95.2	256	35.6		
Indifferent	1	0.14	21	2.9		
**Likelihood of consulting GP about PSA testing in next 12 months PRIOR to seeing decision aid**						
Very Likely	227	31.2	205	28.5	3.10 (df 3)	p = 0.3763
Likely	187	29.6	213	29.6		
Unlikely	244	33.6	239	33.2		
Very Unlikely	69	9.5	63	8.8		
**Likelihood of consulting GP about PSA testing in next 12 months AFTER seeing decision aid**						
Very Likely	219	30.1	237	32.9	12.75 (df 3)	p = 0.0052
Likely	214	29.4	255	35.4		
Unlikely	228	31.3	180	25.0		
Very Unlikely	66	9.1	48	6.7		

## Discussion

### Summary

Our most important results can be summarized as follows: 1) personalising the choice of attributes had a significant effect on the number of men for whom the decision aid generated an opinion in favor of PSA testing; 2) the quality of the decision made (or opinion generated by the aid) using the personalised decision aid was at least as good as, and possibly better than, that made using the fixed decision aid; 3) in the personalised choice group, the significance of overall survival was offset by other attributes including avoiding regret and carer burden, which are not included as attributes in standard prostate screening decision aids. Men were, therefore, choosing to minimize a potential loss as well as maximize the potential health gain associated with PSA screening. Contrary to expectations, avoiding biopsy was not amongst the most important factors for men deciding on PSA screening for either group; and 4) many men for whom the opinion of the aid was against PSA screening stated, when asked directly, that they still intended to undergo screening. The use of the decision aid in both fixed and personalised forms did not, therefore, have a predictable impact on intended prostate cancer screening behavior.

### Limitations

The main purpose of this study was to test the impact of personalising a decision aid developed specifically for prostate cancer screening. The generalizability of the results are limited by (a) the disease specific nature of the study, and (b) that group averages do not necessarily correlate with the individual’s screening choice. A further limitation is that we were unable to follow up actual screening behavior of the participants after completing the survey.

### Practical implications

#### Implications for decision support

Our finding that men in the personalised choice group were often just as concerned, or more concerned about factors such as avoiding regret and carer burden as they were about were avoiding loss of life, needless biopsy or the burdens of treatment, provides support for extending the set of regular criteria used in such decision aids, and for allowing users to select those that are most significant to them. After all, decision aid-guided shared decision-making will only have resonance if clinicians and patients know which factors are most salient to the patient in any given context. This view is further supported by the finding that personalising the prostate screening decision aid had no adverse impact on self-rated decision quality.

The finding that many men (in both groups) whose use of the aid generated an opinion against PSA testing still stated an intention to undergo screening has implications for the utility of decision aids in general. This is not to say that decision aids are useless, but rather that they are what their name suggests—decision aids—aimed at facilitating communication, informing and assisting people to feel confident in the decisions they make. They are not, and should not be mistaken for, tools that can “change people’s minds” or predict their ultimate intentions and actions.

It is, however, noteworthy that, while the decision aid had little impact on the intention to undergo screening, there was much less of a disjunction between opinions as generated by the decision aid and stated intentions in the personalised choice group than in the fixed attributes group (In the fixed attribute group, 5% of men had an opinion generated to have PSA testing and 59.5% stated that they were likely or very likely to undergo testing in the next 12 months; in the personalised choice group, 62% of men had an opinion generated to have PSA testing and a similar percentage of 68.3% stated they were likely of very likely to be tested in the next 12 months). This finding suggests that personalising decision aids may create a higher degree of congruence between the opinions generated by decision tools and actual behavior. Moreover, these findings suggest that personalised decision aids might be better tools for facilitating communication and decision-making than fixed attribute aids.

#### Implications for policy

Our findings provide clear justification for further investigation of the merits of personalised decision aids, but their broader policy implications are more complicated. While both evidence-based medicine and patient-centered care emphasize the importance of factoring patient or community preferences into decision-making, neither paradigm explains what to do when these preferences (expressed through decision aid-generated opinions and/or stated intentions) conflict with scientific evidence and policy guidance.

On the one hand, it could be argued that we should accept any preferences that men express, even if these go against current policy recommendations. On the other, while it is desirable to provide health care that is consistent with expressed preferences, this is not the only consideration for those providing care or making health care policy decisions. There are at least three reasons for, on occasions, overriding patient or community preferences.

First, some degree of “soft paternalism” is inevitable in medical and public health practice. While patients and communities are encouraged to make autonomous decisions about treatment, this occurs within parameters of evidence-based “best practice” as determined by clinical and public health practitioners. Second, resource allocation issues cannot be ignored when making health care policy. In the case of PSA screening, it is essential to consider both consumer preferences and the costs to the community of the testing itself and the unnecessary interventions that might result from systematic “over-diagnosis.” Third, patient and community preferences might be strongly expressed and compelling, but they may not be fully “rational.” We know, for example, that people often make decisions on the basis of previous decisions and experiences. Of course decisions based on previous experience should not be dismissed as “irrational,” but evidence of this kind of reasoning adds a layer of complexity to the interpretation of preferences.

These are the reasons why clinicians and policy makers face major challenges in applying population-level evidence to preference-sensitive decisions. Matters are complicated further by the fact that it is very difficult to take away clinical options when they are already funded and/or highly publicized, as in the case of PSA testing and many other screening tests [50, 51].

The policy implications of our findings thus depend on how clinicians and policy makers choose to balance evidence against patient and community preferences in the context of established clinical and public health practices. What seems clear, however, is that decision-making will need to be individualised no matter how apparently compelling a policy directive might be, and this might be aided by the use of personalised decision aids.

#### Conclusion

Personalising a decision-aid for prostate cancer had a significant impact on the opinion generated by the aid regarding PSA screening, on the reasoning underpinning these opinions, and on the congruence between decision aid-generated opinions and stated behavioral intentions, without impacting negatively on self-rated decision quality. Our findings provide strong support for further development and investigation of personalised decision-aids. Our findings also demonstrate the complexity of clinical and public health communication and health-related decision-making, and the need for ongoing reflection on how to accommodate expressed patient and community preferences within the frameworks of evidence-based medicine and public health.

## Supporting Information

S1 File Consort Flow Diagram. (DOCX)

S2 File Consort Checklist. (DOC)

S3 File Trial registration protocol. (PDF)

S4 File Research ethics protocol. (PDF)

S5 File Participant information sheet. (PDF)

S6 File Introductory material (both study arms). (DOCX)

S7 File Fixed arm intervention protocol. (DOCX)

S8 File Personalised arm intervention protocol. (DOCX)

S9 File Concluding material (both study arms). (DOCX)

S10 File Personalising Annalisa. (DOCX)

S11 File My Decision Quality explanation and weightings items. (DOCX)
