# Supplementary material for: The Role of Personalised Choice in Decision Support: A Randomized Controlled Trial of an Online Decision Aid for Prostate Cancer Screening
Source: PLoS One. 2016 Apr 6;11(4):e0152999. doi: 10.1371/journal.pone.0152999 (PMC4822955; doi:10.1371/journal.pone.0152999)
Supplement: S3 File — (PDF) [file pone.0152999.s003.pdf]

ANZCTR
Australian New Zealand Clinical Trials Registry

Questions in **bold** text are mandatory. **(*)**

**Request Number:**	343044
**Current Page:**	Review

Trial from ANZCTR

**Trial ID**	ACTRN12612000723886
**Trial Status:**	Registered
**Date Submitted:**	15/06/2011
**Date Registered:**	6/07/2012
	**Retrospectively registered**

Page 1

**Public title**	A randomised controlled trial of an interactive decision aid for prostate cancer screening
**Study title in 'Participant-Intervention-Comparator- Outcome (PICO)' format**	A randomised controlled trial of an online interactive decision aid applied to prostate cancer screening for men aged 40 to 69 years, assessing decision quality
**Secondary ID [1]**	None
UTN	U1111-1121-9610
Trial acronym	None

Page 2

**Health condition(s) or problem(s) studied:**	
PSA testing for prostate cancer.	
**Condition category:**	**Condition code:**
Public Health	Health promotion/education
Public Health	Epidemiology
Cancer	Prostate

Page 3

**Descriptions of intervention(s) / exposure**	We have developed an online decision aid (‘My Prostate Cancer Screening Annalisa’) for men aged 40 to 69 years. The aid summarises the evidence for the benefits, potential harms and other factors associated with screening and elicits individual participant’s preferences for each factor associated with the decision to screen (the benefits, potential harms and other factors).<br>Responses to these questions are used to generate a personalised decision aid for the user.<br>There are two arms of the trial:<br>Control arm: Users are asked to indicate how important 5 attributes of prostate cancer screening (benefits and potential harms) are to them; these were pre-installed by researchers based on literature (RCT data) and a pilot study (i.e. the attributes are fixed). This arm is referred to as My Prostate Cancer Screening Annalisa: Fixed Attributes.<br>Intervention arm: Users are asked to choose the attributes they would like to include in their decision aid, from a total of 10 attributes. That is, users pick their own attributes. This arm is called My Prostate Cancer Screening Annalisa: You Choose. The control decision aid takes about 15 minutes to complete. The intervention decision aid takes about 18 minutes to complete.
**Intervention Code:**	Early detection / Screening
**Intervention Code:**	Prevention
**Comparator / control treatment**	The comparator is the ‘fixed attribute’ interactive decision aid.
**Control group**	Active

Page 4

**Primary Outcome:**	Primary outcome: Decision quality as measured by "MyDecisionQuality" an eight point scale that encompasses dimensions of making a high quality health decision.
***Timepoint**:*	Baseline (after completion of interactive decision aid).

**Secondary Outcome:**	Secondary Outcome 1: The proportion of the intervention and control group who indicate they are more likely to visit their GP to discuss PSA testing after interacting with either the 'Pick Your Own' (PYO) or Fixed Attribute (control group) decision aids. (The PYO version is where participants choose, from a list of 10 attributes, the ones they think are important to them). This will be assessed in the online questionnaire by asking participants to indicate how likely they are to see their GP about PSA testing before and after they use the appropriate decision aid.
Timepoint:	After interaction with the decision aid.

Page 5

**Key inclusion criteria**	40-69 year old men living in Australia who have access to the internet and a reasonable command of the English language.
**Minimum age**	40 Years
**Maximum age**	69 Years
**Gender**	Males
**Healthy volunteers?**	Yes
**Key exclusion criteria**	No access to the internet, insufficient English, younger than 40 years or older than 69 years.

Page 6

**Study type**	Interventional
**Purpose of the study**	Educational / counselling / training
**Allocation to intervention**	Randomised controlled trial
Describe the procedure for enrolling a subject and allocating the treatment (allocation concealment procedures)	Global Market Insite, Inc. (GMI) will recruit 1,398 participants from their panel of 130,000 Australians. Eligible participants who agree to be in the study will be randomly allocated to one of the two arms of the study. This is done centrally by computer.
Describe the methods used to generate the sequence in which subjects will be randomised (sequence generation)	For each of the three age strata simple randomisation is performed using a randomisation table created by computer software (i.e. computerised sequence generation).
Masking / blinding	Open (masking not used)
Who is / are masked / blinded (choose all that apply)	
Assignment	Parallel
Other design features	
Type of endpoint (s)	Efficacy
Statistical Methods/Analysis	

Page 7

Phase	Not Applicable
**Anticipated date of first participant enrolment**	9/06/2011
Date of first participant enrolment	
Anticipated date last participant recruited/enrolled	
Actual date last participant recruited/enrolled	
**Target sample size**	1398
**Recruitment status**	Completed

Recruitment in Australia

**Recruitment state(s)**	NSW,VIC,ACT,QLD,SA,WA,NT,TAS

Recruitment outside Australia

Page 8

**Funding Source:**	Government body
**Name:**	National Health and Medical Research Council (NHMRC)
**Address:**	Level 1<br>16 Marcus Clarke Street<br>Canberra ACT 2601
**Country:**	Australia
**Primary Sponsor**	Individual
**Name:**	Professor Glenn Salkeld
**Address:**	A27 - Edward Ford Building<br>The University of Sydney<br>NSW 2006 Australia
**Country:**	Australia
**Secondary Sponsor:**	None
**Name:**	
**Address:**	
**Country:**	
Other Collaborator:	Individual
Name:	Dr Michelle Cunich
Address:	Sydney Medical School<br>Edward Ford Building (A27)<br>University of Sydney, NSW 2006
Country:	Australia
Other Collaborator:	Individual
Name:	Professor Emeritus Jack Dowie
Address:	Tavistock Place, London School of Hygiene and Tropical Medicine<br>London<br>15-17 Tavistock Place<br>WC1H 9SH UK
Country:	United Kingdom
Other Collaborator:	Individual
Name:	Dr Kirsten Howard
Address:	Sydney Medical School<br>Edward Ford Building (A27)<br>University of Sydney, NSW 2006
Country:	Australia
Other Collaborator:	Individual
Name:	Associate Professor Manish Patel
Address:	Westmead Hospital<br>PO Box 533<br>Wentworthville, NSW<br>2145
Country:	Australia
Other Collaborator:	Individual
Name:	Professor Graham Mann
Address:	Westmead Millennium Institute for Medical Research<br>PO Box 533<br>Wentworthville, NSW<br>2145
Country:	Australia

Page 9

**Has the study received approval from at least one Ethics Committee?**	Yes
Ethics Committee name:	University of Sydney Human Research Ethics Committee
Address:	Level 6<br>Jane Foss Russell Building (G02)<br>The University of Sydney,<br>NSW 2006
Country:	Australia
Approval Date:	02/06/2011
Submitted Date:	31/03/2011
HREC:	05-2011/13712
Brief summary	This study aims to evaluate an online interactive decision aid for prostate cancer screening. Who is it for? You can join this study if you are a male aged 40-69 years who lives in Australia. You must have access to the internet and a reasonable command of the English language. Trial details Participants in this trial will complete an online decision aid for prostate cancer screening. This easy to use interactive decision aid asks men to rate the importance of factors that are relevant to making a high quality

	decision, and to weigh up the potential benefits (e.g. avoiding the potential loss of lifetime by early detection of prostate cancer) and harms (e.g. false positive test results and unnecessary treatments) of PSA testing for prostate cancer. Participants will be randomly (by chance) assigned to one of two groups. One group will complete the 'Fixed Attributes' version of the decision aid and the other group will complete the 'You Choose' version. The usefulness of the decision aid will be assessed via a series of questions asking the respondents to rate aspects of the quality of the decision about prostate cancer screening.
Trial website	
Trial related presentations / publications	
Public Notes	

Page 10

Principal Investigator

Title:	
Name:	
Address:	
Country:	
Tel:	
Fax:	
Email:	

Contact person for public queries

Title:	
Name:	Professor Glenn Salkeld
Address:	Sydney Medical School Edward Ford Building (A27) University of Sydney, NSW 2006
Country:	Australia
Tel:	+61 2 9036 9262
Fax:	+61 2 9036 9019
Email:	glenn.salkeld@sydney.edu.au

Contact person for scientific queries

Title:	
Name:	Professor Glenn Salkeld
Address:	Sydney Medical School Edward Ford Building (A27) University of Sydney, NSW 2006
Country:	Australia
Tel:	+61 2 9036 9262
Fax:	+61 2 9036 9019
Email:	glenn.salkeld@sydney.edu.au

Contact person responsible for updating information

Title:	
Name:	Dr Michelle Cunich
Address:	Sydney Medical School Edward Ford Building (A27) University of Sydney, NSW 2006
Country:	Australia
Tel:	+61 2 9351 8959
Fax:	+61 2 9351 7420
Email:	michelle.cunich@sydney.edu.au
