# Supplementary material for: The Role of Personalised Choice in Decision Support: A Randomized Controlled Trial of an Online Decision Aid for Prostate Cancer Screening
Source: PLoS One. 2016 Apr 6;11(4):e0152999. doi: 10.1371/journal.pone.0152999 (PMC4822955; doi:10.1371/journal.pone.0152999)
Supplement: S4 File — (PDF) [file pone.0152999.s004.pdf]

HREC OFFICE USE		
**HUMAN RESEARCH ETHICS COMMITTEE NUMBER:**		
**Meeting Date:**		
**Chief Investigator/Supervisor's Surname:**		
**Student's Surname:**		
**Agenda Category:**	Honours	
	Masters	
	PhD	
	Grants Awarded	
	Small Grants Awarded	
	General	

ETHICS AND PRIVACY APPLICATION FORM FOR RESEARCH INVOLVING HUMANS

INSTRUCTIONS FOR ALL SUBMISSIONS

	Y	N
Original Application signed [all signatures required before submitting]	X	
10 copies of the signed Original Application plus a soft PDF copy	X	

Please (X) to indicate either "Y" or "N" that the following documents are attached to the Original and Copies:

	Y	N
Participant Information Statement (s)	X	
Consent Form (s)	X	
Copy of questionnaire(s), survey questions, interview topics to be covered etc.	X	
Research references	X	
Recruitment advertisement / circular	X	
Evidence of permission to conduct research in other locations	X	
One copy of the grant application with appropriate clearance forms as requested by the Research Office	X	

Please Note: Each question on this form has instructions and links to relevant documents and guidelines on how to answer that particular question as hidden text. To show the text with the hidden text effect, click symbol "¶" (**Show/Hide)** (situated next to the **"Zoom"** button) on the "**Standard"** toolbar. When hidden text is shown it is marked with a dotted underline. This text will not be seen on the printed version.

Please note the following:

1. This application must be completed electronically or typewritten
2. Complete all sections except those specifically not applicable
3. Use lay terms wherever possible
4. Do not alter the order of questions or layout of the application form
5. "Y" signifies Yes, "N" signifies No, and "N/A" signifies Not applicable
6. Some "Y"/"N" boxes have been reversed so take care in answering the questions
7. HREC refers to Human Research Ethics Committee

SECTIONS:		Page
Section 1:	Administration	3
Section 2:	Nature of Research	6
Section 3:	Participants and Recruitment	7
Section 4:	Privacy	10
Section 5:	Collection and Dissemination of Results	13
Section 6:	Risks and Benefits	15
Section 7:	Participant Information and Consent	16
Section 8:	Conflict of Interest and Other Ethical Issues	17
Section 9:	Description of Project	18
Section 10:	Field Based Research or Research Conducted Outside Australia	20
Section 11:	Declaration of Researchers	22
	Checklist	23

This form has been prepared in collaboration between Ms G Briody, Associate Professor M Grimm, Professor A Lloyd, Associate Professor J Watson and Ms M Wright of the Human Research Ethics Committees (HRECs) of the Universities of New South Wales and Sydney.

SECTION 1: ADMINISTRATION

This section is obligatory

1.1 (a) Full project title

A randomised trial of an interactive decision aid for prostate cancer screening.

(b) Short name by which the project will be known

Prostate cancer screening interactive decision aid project

(c) Name of Chief Investigator

Professor Glenn Salkeld

(d) Provide a brief summary of the project in lay language (approximately 100 words)

Men face a difficult task in weighing up potential benefits (avoiding the potential loss of lifetime due to a premature prostate cancer related death) and harms (such as false positive test results, perhaps an unnecessary biopsy and potential side effects of treatment such as impotence, urinary and bowel incontinence). Decision aids have been shown to be effective in helping patients make informed health choices. This web-based decision aid for prostate cancer screening asks men to rate the importance of factors that are relevant to making a high quality decision, to weigh up the benefit, harms and other factors of PSA testing using a single computer screen, easy to use interactive decision aid. The usefulness of the decision aid will be assessed via a series of questions asking the respondents to rate aspects of the quality of the decision about prostate cancer screening.

(e) Outline the academic/scientific merits of this study (including potential contributions to the body of knowledge and methodological rigor) (approximately 100 words)

This study will examine the views and attitudes of Australian men regarding a new web-based decision aid for prostate cancer screening called *My Prostate Cancer Screening Annalisa* (abbreviated to MyProstScreenAL). Annalisa is the name given to the software platform that supports the decision aid. MyProstScreenAL is a multi criteria decision analytic approach to combining the best available evidence on the potential benefits and harms of prostate specific antigen (PSA) testing with individual's preferences for those attributes of screening. The product of the evidence (the chance that an event or outcome of screening will occur) and individual's preferences generates a ranked list, from best to worst, of the available options. Thus it is the men's own preferences for each aspect of screening and its outcomes which, combined with the scientific evidence, provides them with the best platform on which to make an informed choice about PSA testing for themselves. This study will provide information on which factors are most important to men in making a high quality decision about PSA testing and will assist the future development of an interactive decision aid for prostate cancer screening – one that would be publicly available to anyone with internet access.

1.2 Indicate the institutional ethics committee that you consider to be the primary one for this project. (In general, if the Chief Investigator is a University employee, then the University should be considered to be the primary site. If the Chief Investigator or participants are from a health care service, then the Area Health Service ethics committee should be considered as the primary site.)

The University of Sydney

1.3 (a) Has this project already been submitted to any other HREC(s)? [X] **N** [ ] **Y**

(b) Will this project be submitted to any other HREC(s)? [X] **N** [ ] **Y**

If you answered YES to (a) or (b), give the name of the HREC(s), and indicate the status of the application at each (i.e., submitted, approved, deferred or rejected). Attach copies of the correspondence with each of the other HREC(s).

Please do not submit to more than one HREC concurrently.

1.4 List the following details of the Chief Investigator/Supervisor, any Co-Researcher(s), Associate Researcher(s) and Student(s).

Chief Investigator/Supervisor

Name	Glenn Salkeld
Title	Prof
Qualifications	B.Bus, G Dip Health Economics, MPH, PhD
Positions held: employed, conjoint/adjunct/visiting	Professor of Public Health and Head of School<br>School of Public Health, University of Sydney
Full mailing address (including building number)	Edward Ford Building (A27)<br>University of Sydney, NSW 2006
Telephone	02 9036 9262
Fax	02 9036 9019
E-mail	glenn.salkeld@sydney.edu.au

Co-Researcher(s), Associate Researcher(s), Student(s) or other Personnel involved in the study (If appropriate indicate for each named person whether they are University staff, student or neither). If the named person is a student, nominate (in the Qualifications section) the degree for which he/she is enrolled.

Name	Michelle Cunich
Title	Dr
Qualifications	B.Ec, M.Ec, PhD (Ec.)
Positions held: employed, conjoint/adjunct/visiting	Research Fellow
Full mailing address (including building number)	Edward Ford Building (A27)<br>University of Sydney, NSW 2006
Telephone	02 9351 8959
Fax	02 9351 7420
E-mail	michelle.cunich@sydney.edu.au

Name	Jack Dowie
Title	Prof
Qualifications	PhD
Positions held: employed, conjoint/adjunct/visiting	Professor Emeritus of Health Impact Analysis
Full mailing address (including building number)	London School of Hygiene and Tropical Medicine<br>London WC1H 9SH UK
Telephone	+44 (0)20 7927 2034
Fax	+44 (0)20 7927 2701
E-mail	Jack.Dowie@lshtm.ac.uk

Name	Kirsten Howard
Title	Dr
Qualifications	BSc(Hons1), MAppSc, MPH, MHlthEc, PhD
Positions held: employed, conjoint/adjunct/visiting	Associate Professor<br>School of Public Health, University of Sydney
Full mailing address (including building number)	Edward Ford Building (A27)<br>University of Sydney, NSW 2006
Telephone	02 9351 2587
Fax	02 9351 7420
E-mail	kirsten.howard@sydney.edu.au

Name	Manish Patel
Title	Associate Professor
Qualifications	MBBS MMed PhD FRACS
Positions held: employed, conjoint/adjunct/visiting	Associate Professor<br>Faculty of Medicine, University of Sydney
Full mailing address (including building number)	Westmead Clinical School
Telephone	02 9633 2088
Fax	02 9633 3672
E-mail	mpatel@med.usyd.edu.au

Name	Graham Mann
Title	Professor
Qualifications	PhD
Positions held: employed, conjoint/adjunct/visiting	Professor<br>Faculty of Medicine, University of Sydney
Full mailing address (including building number)	Westmead Millennium Institute for Medical Research (C24)<br>Westmead Hospital and University of Sydney
Telephone	02 9845 9056
Fax	02 9845 9102
E-mail	graham_mann@wmi.usyd.edu.au

Insert additional boxes if necessary.

1.5 Who is the nominated Contact Person (from those listed in 1.4 above) for this protocol?

Name	Telephone Number	Email
Glenn Salkeld	02 9036 9262	glenn.salkeld@sydney.edu.au

1.6 Who is the person preparing this document?

Name	Telephone Number	Email
Michelle Cunich	02 9351 8959	michelle.cunich@sydney.edu.au

1.7 Are there students involved in this project? [X] N [ ] Y

If you answered YES, indicate the number of students covered by this study and the degrees which this study will contribute towards (i.e., Honours, Masters, PhD, etc.) **If the names are already known please include them.**

1.8 (a) Indicate the proposed date of commencement of the project.
Projects may not commence without the prior written approval of the HREC.

Date April 22 2011

(b) Indicate the proposed completion date of the project.

Date April 2012

1.9 Indicate all location(s) at which the research will be undertaken.

The University of Sydney.

1.10 (a) Has this protocol received research funding/contracting or is this submission being made as part of an application for research funding/contracting? [X] N [ ] Y

If you answered YES, list the funding/contracting bodies to which you have submitted, or intend to submit, this project. Attach a copy of the grant application(s), contract(s) or similar agreement(s).

(b) What is the outcome of these funding/contracting application(s) (please tick the appropriate box)

RIMS_ID: **Funding/Contracting body 1:** [ ] [ ] [ ]

		Approved	Pending	Refused
RIMS_ID:	Funding/Contracting body 2:	☐	☐	☐
		Approved	Pending	Refused
RIMS_ID:	Funding/Contracting body 3:	☐	☐	☐
		Approved	Pending	Refused

(c) Will this study still be undertaken if funding is not successful? ☐ **N** ☐ **Y**

(d) If the title of the project submitted for funding is different from that listed under Q1.1(a), state it below.

Proceed to Section 2.

SECTION 2: NATURE OF RESEARCH
(refer to the National Statement on Ethical Conduct in Research Involving Humans, p. 23-45)

This section is obligatory

2.1 The nature of this project is most appropriately described as research involving:- (more than one may apply):

	N	Y
– behavioural observation	X	
– self-report questionnaire(s)		X
– interview(s)	X	
– qualitative methodologies (e.g. focus groups)	X	
– psychological experiments	X	
– epidemiological studies	X	
– data linkage studies	X	
– psychiatric or clinical psychology studies	X	
– human physiological investigation(s)	X	
– biomechanical device(s)	X	
– human tissue (see Section 11 – Medical Form)	X	
– human genetic analysis (see Section 11)	X	
– a clinical trial of drug(s) or device(s) (see Section 12)	X	
– Other (please specify in the box below)	X	

Proceed to Section 3.

SECTION 3: PARTICIPANTS AND RECRUITMENT
(refer to the National Statement on Ethical Conduct in Research Involving Humans, p. 25-34)

This section is obligatory

3.1 (a) What is the age range of all participants involved in this study?

All participants will be men aged 40 to 69 years

(b) If the participants include children (defined by statute for this purpose as anyone under 18) has a Prohibited Employment Declaration Form for the researchers ("criminal record check") been lodged with the University or hospital? (see http://www.kids.nsw.gov.au/check/) ☐ **Y** ☐ **N**

If you answered NO, give reasons why not.

3.2 Are the participants:-
(more than one may apply)

	N	Y
– **in a teacher–student relationship with the researchers or their associates?**	X	
– **in an employer–employee relationship with the researchers or their associates?**	X	
– **in any other dependent relationship with the researchers or their associates?**	X	
– **wards of the state?**	X	
– **prisoners?**	X	
– **refugees?**	X	
– **members of the armed services?**	X	
– **mentally ill?**	X	
– **intellectually impaired?**	X	
– **unconscious or critically ill patients?**	X	
– **under the Guardianship Act 1987 (as amended)?**	X	
– **in a doctor–patient relationship or a health giver–receiver relationship with the researchers or their associates?**	X	
– **Aboriginal or Torres Strait Islanders?**	X	

If you answered YES to any of the above, provide details.

3.3 (a) **What is the sample size for the study? Comment on how this sample size will allow the aims of the study to be achieved.**

We aim to have complete data for 1,398 participants consisting of 699 completed responses for arm 1 (‘Fixed attribute’ comparator group) of the study and 699 completed responses for arm 2 (‘Pick Your Own’ attributes i.e. respondent generated attributes). The primary outcome of the study is the quality of decision making. The sample size is based on the number needed to detect a 0.15 difference in the mean decision quality scores between respondents in the two arms of the study.

Further details:
The primary outcome measure for the study is the quality of their decision about PSA testing made using the decision aid, which will be self rated on a continuous scale from 0 to 1. We assume that the response within each subject group is normally distributed with standard deviation 1 (standardized). If the true difference in the arm 2 (considered to be the *experimental* group) and arm 1 (considered to be the *control* group) decision quality means is 0.15, we will need to study 699 arm 2 subjects and 699 arm 1 subjects to be able to reject the null hypothesis that the population means of the arm 2 and arm 1 groups are equal with probability (power) 0.8. The Type I error probability associated with this test of this null hypothesis is 0.05.

(b) **How will the participants be recruited?**
Investigators should note that the initial contact with participants should be at “arm’s length” to avoid real or perceived coercion.

Participants will be recruited through a market research company with extensive experience in running online surveys, Global Market Insite, Inc. (GMI). Initial contact will be made by GMI, inviting men aged 40-69 years to complete our online survey. The participant group, from which the sample will be randomly selected, is men aged 40 to 69 years living in Australia, who are able to complete an online survey written in English. The sample will be drawn from GMI’s consumer panel called ‘GlobalTestMarket’ to which it has recruited approximately 130,000 panelists from Australia. GMI will send invitations via email to their panellists, each contains a unique link – this is standard across all GMI projects. Invited participants will view GMI’s start page, upon clicking the next arrow they will be directed to our study. We will provide GMI with web links for the 2 versions of the survey (Fixed or ‘Pick Your Own’ attributes). Clicking that next arrow will bring up on their screen the first page of the survey. A prompt will ask them to read the Participant Information Sheet before proceeding with answering the survey questions (see Appendix 1). Identifying information (such as names and contact details) of the respondents will not be provided to researchers at the University of Sydney or The London School of Hygiene and Tropical Medicine (LSHTM). Limited information on the socio-demographic characteristics of participants such as age, country of birth and level of education will be collected as part of the online survey.

The sample recruitment will approximate the age distribution of men aged 40-69 years in the Australian population. Participants will be randomised into two distinct groups, with each group to complete one arm of the study (i.e. one survey). The research team will provide GMI with the web links for the surveys. GMI will direct each potential participant to one survey (i.e. Fixed or ‘Pick Your Own’) and he will be able to enter the survey. We have engaged a computer programmer at the University who will set up links that provide updates to GMI regarding interview length (length of time an individual spends on the survey), qualification (that the correct numbers of men randomly selected to each version of the survey are filling in the surveys), screener (men who screen out of the survey) and completion rates. This information will be used by GMI to identify panelists who have completed our surveys (and hence when our quotas are met). The first page of our survey will contain study information (see Appendix 2 Transcript for “Brief message from Professor Salkeld”). Once the participant has completed an online consent (see Appendix 3 – Question 1 on page 2 of the survey) he will be asked to commence the survey.

3.4 (a) **Does recruitment involve a direct personal approach from the researchers to the potential participants?** [X] **N** [ ] **Y**

If you answered YES, explain how the real, or perceived, coercion from researchers for potential participants to enrol has been addressed.

(b) Does recruitment involve the circulation/publication of an advertisement, circular, letter, email letter etc?

X N	Y

If you answered YES, provide a copy. If recruitment involves an advertisement, please indicate where and how often it will be published.

3.5 Will participants receive any reimbursement of out-of-pocket expenses, or financial or other "rewards" as a result of participation?

N	X Y

If you answered YES, what is the amount or nature of the reward and the justification for this?

Whilst participants will not receive any financial or other reward from the University of Sydney, the market research company that will be recruiting our participants, GMI, have a points system whereby MarketPoints are accrued for participation. These points are redeemable for money from GMI. The points represent modest compensation for the half hour or so that participants devote to completing the questionnaire.

3.6 Is the research targeting any particular ethnic or community group? [X] N [ ] Y

If you answered YES, which group is being targeted?

If you answered YES, is there an investigator who is a member of the Particular ethnic or community group? [ ] Y [ ] N

If you answered YES to 3.6, has this project been planned in consultation with a representative of this group? [ ] Y [ ] N

If you answered YES, who have you consulted and how do they represent this group?

If you answered NO, give reasons why you have not consulted.

Proceed to Section 4.

SECTION 4: PRIVACY
Refer to the National Statement on Ethical Conduct in Research Involving Humans, p. 52-53. For health related information refer to the Statutory Guidelines made under the *Health Records and Information Privacy (HRIP) Act 2002 (NSW) Statutory Guidelines on Research* via Privacy NSW HRIP Act and also the NHMRC overview document *The Regulation of Health Information Privacy in Australia*
http://www.nhmrc.gov.au/publications/synopses/nh53syn.htm

This section is obligatory

4.1 Is there a requirement for the researchers to identify, collect, use, or disclose information of a personal nature *(either identifiable or potentially identifiable)* about individuals without their consent?

(a) from Commonwealth departments or agencies? [X] N [ ] Y

(b) from State departments or agencies? [X] N [ ] Y

(c) from other third parties, such as non-government organisations? [X] N [ ] Y

If you answered YES to (a), (b) or (c), state what information will be sought and how many records will be accessed.

4.2 (a) Is there a requirement for the researchers to identify, collect, use, or disclose personal health information about individuals without their consent, which is identifiable or potentially identifiable? [X] N [ ] Y

IF YOU ANSWERED NO, YOU DO NOT NEED TO COMPLETE ANY MORE OF SECTION 4. GO TO SECTION 5

If you answered YES, indicate the reason(s)

- The project involves linkage of data [ ] Y
- Scientific deficiencies would result if de-identified information was used [ ] Y
- Other [ ] Y

Please provide details

4.3 Will the health information that is identifiable or potentially identifiable with respect to individuals be collected, used or disclosed without the consent of the individual(s) concerned? ☐ N ☐ Y

If you answered YES, indicate the reason(s)

- The size of the population involved in the research. ☐ Y
- The proportion of subjects who are likely to have moved or died since the health Information was originally collected. ☐ Y
- The risk of introducing bias into the research, affecting the generalisability and validity of the results. ☐ Y
- The risk of creating additional threats to privacy by having to link information in order to locate and contact subjects to seek their consent of the results. ☐ Y
- The risk of inflicting psychological, social or other harm by contacting subjects with particular conditions in certain circumstances. ☐ Y
- The difficulty of contacting individuals directly when there is no existing or continuing relationship between the organisation and the individuals. ☐ Y
- The difficulty of contacting individuals indirectly through public means, such as advertisement and notices. ☐ Y
- Other ☐ Y

Please provide details

4.4 Was this research the primary purpose of collecting the health information? ☐ Y ☐ N

If you answered YES, you do not need to complete any further questions in Section 4. Go to Section 5

If you answered NO, please provide details

4.5 Would the subjects have expected the researchers to use or disclose their health information for the purposes of this project? ☐ Y ☐ N

Please provide details

4.6 Explain why the collection, use or disclosure of this information is in the public interest, and why the public interest in the project substantially outweighs the public interest in the protection of privacy.

Proceed to Section 5.

SECTION 5: COLLECTION OF DATA AND DISSEMINATION OF RESULTS
(refer to the National Statement on Ethical Conduct in Research Involving Humans, p. 52-53)

This section is obligatory

5.1 Will any part of the study involve <u>recordings</u> using audio tape, film/video, or other electronic medium ?
If you answered YES, what is the medium and how it will be used?

N	Y
[ ]	[X]

The study will involve prerecorded audiovisual clips that demonstrate the purpose and use of the interactive decision aid. These clips have been recorded by the study CI Prof Glenn Salkeld. The survey itself will be completed entirely online.

5.2 Does your research involve the <u>secretive</u> use of photographs, tape-recordings, or any other form of record-taking?
If you answered YES, provide details and a justification for the secrecy.

N	Y
[X]	[ ]

5.3 (a) How will the results of the study be disseminated (e.g. via publication in journals and presentations in scientific meetings)?

The results of this study will be presented at local and international conferences and published in peer-reviewed journals.

(b) How will feedback be made available <u>to participants</u> (e.g. via a lay summary or newsletter)?

Please cross (X) the appropriate box:

	**One (1) Page Lay Summary**
	**Written Transcripts**
	**Newsletter**
	**Report**
X	**Web-based Feedback**
	**If NO feedback will be given, provide details below**

Participants will be able to view their results 'in real time' and will be offered the chance to print out their results as they complete the survey.

5.4 How will the confidentiality of the data, including the identity of participants, be ensured during collection and dissemination?

No respondent identifying information will be provided to researchers at the University of Sydney or the LSHTM by GMI. The individual responses to the survey questions will be stored securely in a section of the University server which is password protected and only accessible by the Chief Investigator, Professor Glenn Salkeld (the Administrator for this web site). The server can only be accessed by the Administrator, and data files (results files) will be password-protected for access only by the researchers. Since there is no direct contact between the researchers and participants, anonymity is further assured. All analyses and results will therefore be conducted on de-identified data.

5.5 Is there any possibility that information of a personal nature could be revealed to persons not directly connected with this research?
If you answered YES, provide details.

X	
**N**	**Y**

5.6 (a) What is the proposed storage location of, and access to, materials collected during the study (including files, audiotapes, questionnaires, videotapes, photographs)?

Please cross (X) the appropriate box:

	**Chief Investigator/Supervisor's Office**	**Room No.**		**Building No.**	
	**Faculty / Departmental Office**	**Room No.**		**Building No.**	
X	**Other** (Please provide details below)				

Data files will be securely stored on the University Server and will be protected by network and file-specific passwords, along with the University's firewall and security systems.

(b) On completion of the study, where will the materials that were collected during the study (including files, audiotapes, questionnaires, videotapes, photographs) be stored?

Please cross (X) the appropriate box:

	**Chief Investigator/Supervisor's Office**	**Room No.**		**Building No.**	
X	**Faculty / Departmental Office**	**Room No.**	123	**Building No.**	A27
	**Other** (Please provide details below)				

All materials will be securely stored on the Medical Faculty's server and will be protected by network and file-specific passwords, along with the University's firewall and security systems.

(c) Specify how long materials collected during the study (including files, audiotapes, questionnaires, videotapes, photographs) will be retained after the study, and how they will ultimately be disposed of.

Please ensure that the period of data retention stated here is appropriate to the nature of the proposed study. If the project involves clinical trial(s), the data should be kept for a minimum of 15 years (please refer to http://www.fda.gov/oc/ohrt/irbs/websites.html). If the projects do not involve clinical trial(s), the data should be kept for a minimum of 7 years after which time the data may be disposed of. *(Please also refer to National Statement on Ethical Conduct in Research Involving Humans, 12.11 for further requirements*).

Please cross (X) the appropriate box:

	**15 years for clinical trials**
X	**7 years**
	**Other** (Please provide details below)

Please cross (X) the appropriate box/es:

	**Paper / CD / DVD Shredding**
	**Audio / Video Tapes Erased**
X	**Other** (Please provide details below)

Files will be deleted from the Medical Faculty server after 7 years. The IT department will be involved to ensure that deletion is complete and files are nor retrievable from any source.

Proceed to Section 6.

SECTION 6: RISKS AND BENEFITS
(refer to the National Statement on Ethical Conduct in Research Involving Humans, p. 51)

This section is obligatory

6.1 (a) Could participation in the research adversely affect the participants? [X] N [ ] Y

If you answered YES, complete 6.1 (b) and 6.1 (c). If you answered NO go to 6.2

(b) Could the research induce any psychological distress in the participants? [X] N [ ] Y

(c) Could the research cause any physical harm to the participants?
(e.g. from physically invasive procedures or from drug administration, etc) [X] N [ ] Y

If you answered YES to (b) or (c) describe the aspect(s) of the research and all the risks involved. Indicate the rate at which these risks are expected to occur. Indicate what facilities and trained personnel are available to deal with such psychological or physical problems.

6.2 Will the true purpose of the research be concealed from the participants? [X] N [ ] Y

If you answered YES, outline the rationale and provide details for the concealment.
Provide details of the debriefing. (If you do not intend to debrief, give reasons why not).

6.3 Are you doing research on patients (i.e. subjects receiving health care)? [X] N [ ] Y

If you answered YES, list the procedures/techniques which would <u>not form part of routine</u> clinical management.

6.4 Is this research expected to benefit the participants directly or indirectly? [ ] N [X] Y

If you answered YES, provide details.

Participants who are contemplating having a PSA test for prostate cancer may find the decision aid helps them in making that decision.

Proceed to Section 7.

SECTION 7: PARTICIPANT INFORMATION AND CONSENT
(refer to the National Statement on Ethical Conduct in Research Involving Humans, p.12-13, p.28-29, p. 40-42, p.44-45, p.47-50, p.54)

This section is obligatory

7.1 Will a Participant Information Statement be provided? [X] Y [ ] N

7.2 Will written consent be obtained? [ ] Y [X] N

If you answered NO to either 7.1 or 7.2, give reasons why not.

Participants will be asked on line if they consent to completing the survey and subsequent completion and submission of the survey itself will provide further proof of their consent.

7.3 In the case of participants who may not be fluent in English or who have difficulty understanding English, will arrangements be made to ensure comprehension of the Participant Information Statement and Consent Form? [ ] Y [X] N

If you answered NO, give reasons. If you answered YES, what arrangements have been made?

As we are developing and testing a brand new interactive decision aid for prostate cancer screening we need to demonstrate the concept and application first (with men who understand written English) before adapting the decision aid appropriately for men from different cultural backgrounds.

7.4 (a) Do the Participant Information Statement and Consent Form have:-

	Y	N
– **the first page of the Participant Information Statement and Consent Form printed on appropriate institutional letterhead?**	X	
– **the title of the project on every page, including the Revocation of Consent? (if one is required) (Use a short title as appropriate)**	X	
– **the page numbers expressed as page 1 of .., 2 of .., 3 of .. etc?**	X	
– **an assurance that participation is voluntary and participants are permitted to withdraw from the project at any time without penalty?**	X	
– **the name and telephone number of an appropriate researcher?**	X	
– **a telephone number, fax number and E-mail address for the HREC, should a participant wish to make a complaint about the conduct of the research project?**	X	

(b) How has the possibility of withdrawal from the study been addressed in the Participant Information Statement and Consent Form?

After reading the online participant consent statement (See Appendix 4 – Question 1 on page 2 of the survey), potential participants will be able elect not to proceed to the online survey. In addition, respondents who do not proceed with the completion of the survey will be treated as though they have withdrawn consent and their responses will not be used.

Participants are also advised in the Participant Information Statement (See Appendix 2) that they may discontinue participation in the study at any time.

Proceed to Section 8.

SECTION 8: CONFLICT OF INTEREST AND OTHER ETHICAL ISSUES
(refer to the National Statement on Ethical Conduct in Research Involving Humans, p. 51–54, Appendix 2)

This section is obligatory

8.1 Are any "conflict of interest" issues likely to arise in relation to this research? [X] N [ ] Y

If you answered YES, provide details.

8.2 Do the researchers have any affiliation with, or financial involvement in, any organisation or entity with direct or indirect interests in the subject matter or materials of this research? [ ] N [X] Y
(Note that such benefits must be declared in the Participant Information Statement.)
If you answered YES, provide details.

Prof Jack Dowie is a part owner of the decision aid software (Annalisa). Prof Dowie will derive no financial gain from his participation in this study. We will declare this on the Participant Information Statement. None of the other CIs has any affiliation or financial involvement with the subject matter or materials of this research.

8.3 Do the researchers expect to obtain any direct or indirect financial or other benefits from conducting this research? [X] N [ ] Y
(Note that such benefits must be declared in the Participant Information Statement.)
If you answered YES, provide details.

8.4 (a) Have conditions already been imposed upon the use (eg. publication), or ownership of the results (eg. scientific presentations) or materials (eg. audio-recordings), by any party other than the listed researchers? [X] N [ ] Y

(b) Are such conditions likely to be imposed in the future? [X] N [ ] Y

If you answered YES to (a) or (b), provide details.

Proceed to Section 9.

SECTION 9: DESCRIPTION OF PROJECT
(refer to the National Statement on Ethical Conduct in Research Involving Humans, p. 13)

This section is obligatory

9.1 Describe the project using lay terms wherever possible, including the aims, hypotheses, research plan and potential significance. Where relevant, provide the projected number, sex, and age range of participants (including inclusion/exclusion criteria). You must satisfy the HREC that the study is scientifically valid (include at least four (4) research references) and conducted in accordance with the accepted ethical principles governing research involving humans.

The description must be no longer than 2 pages and must be in a font size of at least 10 points

Background

Recent evidence suggests that prostate cancer screening using PSA testing offers population benefits in terms of reducing prostate cancer specific mortality, with a recent European trial reporting a relative risk reduction of 0.20 in men who undergo PSA testing and an absolute risk reduction of less than 1 prostate cancer death avoided per 1000 men screened over 9 years.. However this RCT and others (US and Swedish trials) also report evidence of harms: men who participate in screening have a significantly higher likelihood of experiencing a false positive PSA test result (a positive PSA test result but a negative biopsy result - a so called 'false alarm'), overdetection (diagnosis of prostate cancers that would not have become clinically visible during the man's lifetime), and harms from treatment such as incontinence, impotence and bowel functioning problems [1-4].

The benefits and harms of prostate cancer screening need to be finely balanced by men, and the choice to screen or not screen is likely to be driven by how individuals weigh up these benefits and harms. Decision aids are one way of helping men clarify their values and the importance of various aspects of screening.

We have created an online interactive decision aid for prostate cancer screening, called *My Prostate Cancer Screening Annalisa* (abbreviated to MyProstScreenAL), using software known as Annalisa© [5]. Annalisa is a decision aid template based on the decision theory of Multi-Criteria Decision Analysis (MCDA). This theory recognizes that there are often multiple and competing criteria which drive decision making. Methods applied within the MCDA framework assess the individual's 'trade off' between criteria and allow for options to be ranked according to the individual's assessment of the criteria.

MyProstScreenAL combines best available evidence (sources are RCTs discussed above) on the benefits and harms of prostate cancer screening (prostate specific antigen [PSA] testing) with the individual's preferences for different aspects of screening (attributes). The product of the evidence (the chance that an event or outcome will occur) and the individual's preferences generates a rated and ranked list of the options. Thus it is the man's own preferences for each aspect of screening and its outcomes which, combined with scientific evidence, provides him with the best platform on which to make an informed choice about screening.

We have already introduced Annalisa© to the research community by developing and piloting a basic decision aid for prostate cancer screening using it (called *ALProst*) [6]. This decision aid incorporated evidence on both benefits and harms of prostate cancer screening from published RCTs. Ten GPs piloted the aid. Most GPs agreed/strongly agreed with positive statements about the ease with which they could use Annalisa (seven GPs), and understand the information in, and format of, Annalisa (nine and eight, respectively). Eight agreed/strongly agreed that ALProst would be a useful tool for discussing prostate cancer screening with their patients.

MyProstScreenAL will be evaluated in a random sample of Australian men (aged 40-69 years) via an online survey incorporating the decision aid. An assessment of the individual's decision quality (the main outcome measure for the study) will be obtained via the survey.

Aims

The primary aim of this study is to establish whether Australian men (aged 40-69 years) regard MyProstScreenAL as a useful tool when making a decision about prostate cancer screening, and one that promotes a high quality decision. The specific aims of the study are: (1) Test the null hypothesis that the mean 'high quality decision score' is not significantly different between the two study groups (grp 1 'Fixed attributes' comparator and grp 2 'Pick Your Own' or respondent generated attributes); and (2) Test the null hypothesis that the mean attribute weights are not significantly different between the two study groups. We will also compare (statistically) the risk (age group and family history) and socio-demographic characteristics of respondents who found the aid useful (i.e. indicated they were able to make a high quality decision) and those who did not.

Research Plan

We will undertake a randomised controlled trial of MyProstScreenAL. Men living in Australia (GMI panelists; see below) aged 40–69 years who can read English and have internet access will be eligible.

There are two arms of the MyProstScreenAL survey. Respondents in arm 1 are presented with five attributes which are factors determined to be important in making a decision about prostate cancer screening by GPs in our earlier GP pilot study of Annalisa (University of Sydney HREC approved project, Reference No. 12-2007/10481) and in the literature. The five attributes used in arm 1 are:

LOSS OF LIFETIME: Avoid losing 5-10% of your remaining life expectancy, depending on your age.

NEEDLESS BIOPSY: Avoid having a needless biopsy.

URINARY PROBLEMS: Avoid urinary problems after treatment from prostate cancer.

BOWEL PROBLES: Avoid bowel problems after treatment from prostate cancer.

SEXUAL PROBLEMS: Avoid sexual problems (impotence) after treatment from prostate cancer.

Arm 2 of the study (called ‘Pick Your Own’) differs from arm 1 only in that instead of being presented with the five specified attributes, a list of nine attributes is presented to the respondent, including those five. They are asked to select up to nine to include in the Annalisa for which they will provide weightings and see the scores. The additional four attributes, with labels and definitions, are:

LOSS OF HEALTH: Avoid a reduction of 5 to 10% in your overall health level during your lifetime, where health is as defined and valued by the average member of the population.

OVERDIAGNOSIS: Avoid being correctly diagnosed as having prostate cancer, but where the cancer would not have affected your life or health, so any treatment undergone would have been unnecessary.

TREATMENT BURDEN: Avoid experiencing all the various tests and treatments associated with correctly diagnosed prostate cancer.

REGRET: Avoid experiencing regret, IF you did develop prostate cancer that needed treatment, that you had not had the PSA test and been managed in the light of its result.

Respondents will be asked to adjust the weightings of attributes to reflect their importance to them. Following this, the options (PSA Test or No PSA Test) will be rated and ranked by the decision aid (using MCDA). See

Recruitment

Participants will be recruited via a market research company, Global Market Insite, Inc. (GMI). GMI has extensive experience conducting online sampling and survey fieldwork for university-based clients and thus has a thorough understanding of the processes underlying academic work. It owns and maintains a large database (panel) of over 130,000 Australians, who have indicated their willingness to be involved in online survey research. Potential participants meeting the gender (male) and age criterion (40–69 years) will be identified by GMI, and invited to participate in our study. A total of 1,389 men – 699 men randomised to arm 1 (fixed attributes) and 699 men randomised to arm 2 (respondent picks attributes) – from the panel will complete the study. These men will have been randomly selected by GMI to each arm. The age profile of men in each arm (grouped by 40-49, 50-59 and 60-69 years) will reflect the breakdown of 40-69 year old men in the Australian population.

GMI will send invitations via email to their panellists, each contains a unique link – this is standard across all GMI projects. Respondents will see GMI’s start page, upon clicking the next arrow they will be directed to our study (i.e. straight to the Fixed or ‘Pick Your Own’ survey). Respondents will be able to view the Participant Information Sheet online (see Appendix 1). Although consent from panelists to be invited and participate in online surveys has already been obtained by GMI (see Appendix 4 GMI’s Privacy Policy, downloaded 28 March 2011), we have included a question about consent in our survey (see Appendix 3 –Question 1 in the survey on page 2). The MyProstScreenAL survey consists of 50 questions (23 pages) on PSA testing, the decision aid for prostate cancer screening, decision quality, and personal characteristics. On actual Annalisa screens (see Appendix 5 – print of the Annalisa decision aid for prostate cancer screening, includes roll over information on what the attributes are and sources of data), respondents will be asked to weight the attributes in terms of how important they are to them. The options will then be rated and ranked for them. Following completion of questions about decision quality, respondents will be asked some additional socio-demographic questions such as country of birth and education. Screen content for each section of the MyProstScreenAL survey is provided as in Appendix 4 of this application.

Analysis

Summary statistics will be generated on men’s preferences (weights) for different aspects (attributes) of screening and the best option for them (scores), data collected via the decision aid. Data on the usefulness of the decision aid (i.e. decision quality) and socio-demographic information collected later in the survey will also be summarised.

Comparisons between arm 1 (fixed attributes) and arm 2 (‘Pick Your Own’) will be made on the basis of: (a) mean ‘high quality decision scores’, (b) mean attribute weights; and (c) mean scores on PSA testing decision.

Associations between: (a) weights and socio-demographic characteristics, and (b) scores and socio-demographic characteristics will be examined in regression analysis.

The analysis will provide information about the trade-offs that men make in weighing up the benefits and harms of screening and will address methodological issues relating to the mode of delivery of health decision aid

Proposed Questionnaire Structure See Appendix 3 for questionnaire content (both for Fixed and ‘Pick Your Own’).

Potential significance This study will provide information on which factors are most important to men in making a high quality decision about prostate cancer screening. It will also assist the future development of MyProstScreenAL with the intention of eventually making it publicly available to anyone with internet access

References

1. Schroder FH, Hugosson J, Roobol MJ et al. Screening and prostate-cancer mortality in a randomized European study. N Engl J Med 2009;360(13):1320-8.
2. Andriole G, Grubb RL, Buys SS et al. Mortality results from a randomized prostate-cancer screening trial. N Engl J Med 2009;360(13):1310-9.
3. Hugosson J, Carlsson S Aus, G et al. Mortality results from the Goteborg randomised population bncased prostate cancer screening trial. Lancet Oncology 2010 July 1.
4. Howard K, Barratt A, Mann G, Patel M. A model of the outcomes of PSA screening for low and high risk men: information to support informed choices. Archives of Internal Medicine, 2009; 169(17): 1603-1610
5. http://www.annalisa.org.uk and http://www.cafeannalisa.org.uk. Accessed September 21, 2010.
6. Cunich M, Salkeld G, Dowie J et al. Integrating Evidence and Individual Preferences Using a Web-Based Multi-Criteria Decision Analytic Tool: An Application to Prostate Cancer Screening. The Patient. In Press.

Proceed to section 10.

**SECTION 10: FIELD-BASED RESEARCH (i.e., CONDUCTED OFF CAMPUS OR OUTSIDE A HEALTH SERVICE) INCLUDING RESEARCH CONDUCTED OUTSIDE AUSTRALIA (refer to the National Statement on Ethical Conduct in Research Involving Humans, p.14, p.31-32)**

This section must be completed for all applications involving EITHER field-based research OR research to be carried out in countries outside Australia (eg. in a school, a corporation, a government department an Aboriginal and Torres Strait Islander community or research in a another country).

10.1 Is your research conducted

		N	Y
**(i)**	**Outside Australia**	X	
**(ii)**	**Off Campus**		X
**(iii)**	**In an Aboriginal and Torres Strait Islander Community**	X	
**(iv)**	**In a School**	X	
**(v)**	**In a Corporation**	X	
**(vi)**	**In a Government Department**	X	
**(vii)**	**In a Hospital**	X	

If you answered NO to all of the above, go to Section 11

10.2 Have you obtained formal permission from relevant authorities for entry to the area to carry out research (e. g., national or local government bodies, organisations of local communities)?

Y: [ ] N: [X]

If you answered YES, name the relevant authorities and attach the relevant correspondence.

If you answered NO, give reasons.

Not applicable

10.3 If research is proposed among members of specific organisations, have you sought approval from those organisations (e. g., church groups, national associations, etc)?

Y: [ ] N: [X]

If you answered YES, name the relevant authorities and attach the relevant correspondence or letter of support.

If you answered NO, give reasons.

Not applicable

10.4 Does the research involve individuals or groups of people who are not

[ ] [X]

formally organised (e.g., people living in a village or town, etc)? **N** **Y**

If you answered YES, indicate the context of the research. How will you obtain access to participants? Indicate any ethical issues that you can foresee in this approach.

The context for this study is that any future population based prostate cancer screening program may be offered to Australian men aged 40-69 years. We will obtain access to men in the likely screening target group via GMI's panel for the Australian population. GMI has 10 years of experience in assisting clients in undertaking online research, including academic projects. They deliver 7000+ projects annually. They are leaders in the industry regarding quality assurance procedures.

10.5 Will your research necessarily involve the acquisition of objects of valuable cultural property (e. g., carvings, paintings, etc)?

[X] **N** [ ] **Y**

If you answered YES, give details of arrangements with owners of the property with regard to access to/acquisition of these items, where appropriate.

10.6 Will your research necessarily involve any activities that are likely to be seen by research participants and/or members of their local communities as in conflict with local practices and customs (e.g. regarding religious or ritual participation)?

[X] **N** [ ] **Y**

If you answered YES, provide details.

Proceed to Section 11.

SECTION 11. DECLARATION OF RESEARCHERS

I/we apply for approval to conduct the research. If approval is granted, it will be undertaken in accordance with this application and other relevant laws, regulations and guidelines.

Signature of Chief Investigator or Supervisor

Name ....Glenn Salkeld.................................... Signature: .... Date: ....
(print)

Signature of Associate Researcher(s) or Student(s)

Name ...Michelle Cunich.. Signature: .... Date: ....
(print)

Name ....Jack Dowie... Signature: .... Date: ....
(print)

Name ......Kirsten Howard...................................... Signature: .... Date: ....
(print)

Name .... Manish Patel.................................. Signature: .... Date: ....
(print)

Name ....Graham Mann .... Signature: .... Date: ....
(print)

Signature of appropriate senior officer NOT ASSOCIATED with the research (e.g. Head of School OR appropriate).

After careful consideration and appropriate consultation, I have reviewed the attached HREC application, including the Participant Information Statement and Consent Form. I am satisfied that the scientific merit of this work justifies its being performed and that the information which will be obtained justifies the inconvenience and risks to participants.

Name:...
(print)

Title: ....
(print)

Position: ....
(print)

Signature: .... Date: ....
