# Supplementary material for: The Role of Personalised Choice in Decision Support: A Randomized Controlled Trial of an Online Decision Aid for Prostate Cancer Screening
Source: PLoS One. 2016 Apr 6;11(4):e0152999. doi: 10.1371/journal.pone.0152999 (PMC4822955; doi:10.1371/journal.pone.0152999)
Supplement: S5 File — (PDF) [file pone.0152999.s005.pdf]

THE UNIVERSITY OF SYDNEY

School of Public Health
Sydney Medical School

ABN 15 211 513 464

GLENN SALKELD
HEAD OF THE SCHOOL OF PUBLIC HEALTH

Room 319
Edward Ford Building A27
The University of Sydney
NSW 2006 AUSTRALIA
Telephone: +61 2 9036 9262
Facsimile: +61 2 9036 9019
Email: glenn.salkeld@sydney.edu.au
Web:
http://www.usyd.edu.au/medicine/public-health

Title: A randomised trial of an interactive decision aid for prostate cancer screening.

PARTICIPANT INFORMATION STATEMENT

(1) What is the study about?

You are being invited to participate in a study about how Australian men (aged 40-69 years) weigh up the benefits and potential harms of prostate cancer screening and reach a high quality decision. At the moment, Australia does not have a prostate cancer screening program.

You will be introduced to, and asked to have a go at using, a new online decision aid for prostate cancer screening called *My Prostate Cancer Screening Annalisa* (abbreviated to MyProstScreenAL). Annalisa is the name of the software used to create the interactive decision aid.

The main purpose of the study is to find out what factors are most important to men when making a decision about whether to be screened or not, and what factors promote a high quality decision. Your responses will assist us in the future development of MyProstScreenAL – a decision aid that will be made publicly available to anyone with internet access.

(2) Who is carrying out the study?

The study is being conducted by Professor Glenn Salkeld, Professor of Public Health at the Sydney School of Public Health, University of Sydney; Dr Michelle Cunich, Research Fellow in the Economics of Health at the Sydney School of Public Health, University of Sydney; Professor Jack Dowie, Professor Emeritus of Health Impact Analysis at the London School of Hygiene and Tropical Medicine; Dr Kirsten Howard, Associate Professor in Health Economics at the Sydney School of Public Health, University of Sydney; Associate Professor Manish Patel, Associate Professor of Medicine, Westmead Clinical School and University of Sydney; and Professor Graham Mann, Professor of Medicine at the Westmead Millennium Institute for Medical Research, Westmead Hospital and University of Sydney.

(3) What does the study involve?

This study involves answering some questions about MyProstScreenAL and decision quality. On the following screens, you will be presented with some visual and audio information on how to use MyProstScreenAL. You will be asked to weigh up the benefits and potential harms of prostate cancer screening using the interactive decision aid, and to assess the quality of the decision you made. There are also some brief questions about you at the end of the survey.

(4) How much time will the study take?

The study will take 30 minutes to complete and will be conducted in a location convenient to you. If you have a computer with internet access at home, you can complete the survey in the privacy of your own home. The survey is meant to be completed in one sitting.

(5) Can I withdraw from the study?

Being in this study is completely voluntary - you are not under any obligation to consent and - if you do consent - you can withdraw at any time without affecting your relationship with The University of Sydney.

Being in this study is completely voluntary and you are not under any obligation to consent to complete the survey. Submitting a completed survey is an indication of your consent to participate in the study. You can withdraw any time prior to submitting your completed survey. Once you have submitted your survey anonymously, your responses cannot be withdrawn.

(6) Will anyone else know the results?

All aspects of the study, including results, will be strictly confidential and only the researchers will have access to information that you provide in the survey. A report of the study may be submitted for publication, but individual participants will not be identifiable in such a report.

(7) Will the study benefit me?
Participants who are contemplating having a PSA test for prostate cancer may find the decision aid helps them in making that decision.

(8) Can I tell other people about the study?
Yes. You are very welcome to tell other people about the study.

(9) Declaration of investigator benefit
One of the study investigators, Emeritus Professor Jack Dowie, part owns the intellectual property rights for the Annalisa software. Neither Prof Dowie nor any of the researchers involved in this study will derive a financial benefit from your completion of this survey.

(10) What if I require further information?

When you have read this information, Professor Glenn Salkeld will be available to discuss it with you further and answer any questions you may have. If you would like to know more at any stage, please feel free to contact Professor Glenn Salkeld, Head of the Sydney School of Public Health at the University of Sydney. His telephone number is 9036 9262; email address: glenn.salkeld@sydney.edu.au.

(11) What if I have a complaint or concerns?

Any person with concerns or complaints about the conduct of a research study can contact The Manager, Human Ethics Administration, University of Sydney on +61 2 8627 8176 (Telephone); +61 2 8627 8177 (Facsimile) or ro.humanethics@sydney.edu.au (Email).

This information sheet is for you to keep
