# Supplementary material for: The Role of Personalised Choice in Decision Support: A Randomized Controlled Trial of an Online Decision Aid for Prostate Cancer Screening
Source: PLoS One. 2016 Apr 6;11(4):e0152999. doi: 10.1371/journal.pone.0152999 (PMC4822955; doi:10.1371/journal.pone.0152999)
Supplement: S6 File — (DOCX) [file pone.0152999.s006.docx]

**My Decision Navigator**

**Being screened for possible Prostate Cancer is a serious decision which is ultimately up to you to make.**

**Your response to the following questions will take you to the parts of the interactive decision aid and survey that are relevant to your needs.**

**4** **Have you ever been told by a doctor that you have Prostate Cancer?**

Please select one item from the list.

- [1] Yes
- [2] No
- [3] Don't know

**Thanks for accessing our site, but our decision support tool is designed for people who have not been diagnosed with Prostate Cancer.**

**Please click 'Next' and then the 'Submit your Answers' button on the following screen**

**5** **Have you personally had a PSA test before?**

Please select one item from the list.

- [1] Yes
- [2] No
- [3] Don't Know

**5.1** **How long ago was your only, or most recent, PSA test?**

Please select one item from the list.

- [1] Under 6 months
- [2] 6 to 12 months
- [3] 12 months or more
- [4] Don't know

**6** **At this moment, how likely are you to consult your GP within the next 12 months about having a PSA test?**

Please select one item from the list.

- [1] Very Likely
- [2] Likely
- [3] Unlikely
- [4] Very Unlikely

**My Decision Preparation**

**First, a reminder that you will need to give your *informed consent* to any future decision that you may take in relation to screening and treatment.**

**To help ensure this please click 'Next' and watch a short video in which Professor Salkeld talks about PSA testing for possible Prostate Cancer and introduces the interactive decision aid, known as My Prostate Cancer Screening Annalisa**

**7** **Would you like to see a transcript of what was said?**

Please select one item from the list.

- [1] Yes
- [2] No

Hello, I'm Glenn Salkeld, Professor of Public Health at the University of Sydney. Thanks for your interest in My Prostate Screening Annalisa, an interactive decision aid, which we hope will help you make choices about screening.

Well in 2009 the *New England Journal of Medicine* published a paper on a European trial of PSA testing for prostate cancer. The trial found that screening does save lives. Over a 10 year period, screening prevents about 1 death per 1000 men.

But the trial also found that there are potential harms associated with screening. Some men will have an unnecessary biopsy of the prostate due to a false positive test result (so-called false alarm). We also know that when you treat prostate cancer that can cause problems with urinary function, bowel function and sexual function.

So you have to weigh up the harms and benefits of the PSA test to decide whether or not to screen. In this example, the interactive decision aid, known as My prostate screening Annalisa, shows the process by which you might consider making a choice about screening.

The bottom panel shows the two options - to have a PSA test or not to test - and summarises the evidence on the benefits and harms of each option.

The middle panel, the values, represents the importance that you attach to the benefits versus the harms. In this particular example we have the avoidance of loss of lifetime, avoiding a needless biopsy, avoiding urinary, bowel and sexual problems.

The top panel, the scores, shows which option is best. The score is a straight multiplication of the probabilities in the evidence panel and your importance weights from the middle panel, all summed together. The longer the bar, the better the option.

So where do the numbers come from and what do they mean?
The numbers for the evidence panel come from published studies. Roll the curser over the number and you can see the source of the information and there is a link to the published paper if you'd like to check it further for yourself.

The numbers in the values panel represent the importance that you attach to obtaining the benefits and avoiding each of the harms. Sliding the blue bar to the right indicates that you attach more importance to this attribute. Slide it to the left and it is less important to you. The numbers between 0 and 1 simply represent the importance of each attribute with 0 meaning 'no importance' and 1 representing 'all important'.

By moving the blue bar you are trading off the benefits and harms against each other. Note what happens to the score panel when you move the blue bar in the values panel - the scores panel will change. One option may become more attractive compared to the other. To help you get the most out of My Prostate Screening Annalisa there are additional sections before and after the interactive aid itself. They will help prepare you for using the aid and to judge whether you have arrived at a high quality decision.

OK your task now is to go through the program and tell us whether it helps you make a high quality decision about having a PSA test. Remember to click the 'submit your answers' button at the end of the survey - that's the only way we can record your answers.

**Preparing to make a high quality decision**

**If you are seeking to make a high quality decision it is useful to think a little about the ingredients of a good decision before you start the decision making process.**

**Below you will see a list of things that are commonly regarded as criteria for a high quality decision.**

**Different people will weight these criteria differently, so we ask you to indicate how important each criterion is to YOU.**

**Doing this will**

- **Help you *focus* on what YOU see as important as you go through the decision making process**
- **Enable YOU to quickly *judge* the quality of your decision about PSA testing after you have made it - and decide whether you want to revisit it if you are not satisfied.**

|  |  | **Very Low [1]** | **Low [2]** | **Medium [3]** | **High [4]** | **Very High [5]** |
| --- | --- | --- | --- | --- | --- | --- |
| **8** | OPTIONS  **Importance of being clear about the possible OPTIONS for me and what they involve** |  |  |  |  |  |
| **9** | EFFECTS  **Importance of being clear about the possible EFFECTS and outcomes of each of the options for me** |  |  |  |  |  |
| **10** | IMPORTANCE  **Importance of being clear about the relative IMPORTANCE of the different possible effects and outcomes for me** |  |  |  |  |  |
| **11** | CHANCES  **Importance of being clear about the CHANCES of the different effects and outcomes happening to me, including the uncertainties surrounding the best estimates of them** |  |  |  |  |  |
| **12** | TRUST  **Importance of being able to TRUST the information I was given was the best possible** |  |  |  |  |  |
| **13** | SUPPORT  **Importance of feeling I received the level of SUPPORT and consideration I wanted throughout the decision process, especially in regard to communicating at my level** |  |  |  |  |  |
| **14** | CONTROL  **Importance of feeling I was in CONTROL of my decision to the extent I wished** |  |  |  |  |  |
| **15** | COMMITMENT  **Importance of feeling COMMITTED to acting on the decision taken** |  |  |  |  |  |

**My Decision Aid: MyProstScreenAL**

**Things about you which affect your result**

**16** **What is your age?**

Please select one item from the list.

- [1] 40-49 years
- [2] 50-59 years
- [3] 60-69 years

**17** **How many of your first degree male relatives (father, brothers or sons) have been diagnosed with prostate cancer?**

Please select one item from the list.

- [1] 0
- [2] 1
- [3] 2 or more
- [4] Don't know
