# Supplementary material for: The Role of Personalised Choice in Decision Support: A Randomized Controlled Trial of an Online Decision Aid for Prostate Cancer Screening
Source: PLoS One. 2016 Apr 6;11(4):e0152999. doi: 10.1371/journal.pone.0152999 (PMC4822955; doi:10.1371/journal.pone.0152999)
Supplement: S7 File — (DOCX) [file pone.0152999.s007.docx]

**Things which may be important to you**

**We think five (5) considerations (or criteria) will probably be important to you in deciding whether to have a PSA test and, *if required*, further testing and treatment for prostate cancer**

- **Avoiding LOSS OF LIFETIME because of Prostate Cancer**
- **Avoiding a NEEDLESS BIOPSY as a result of a (false) positive PSA test

  The other three concern outcomes *that might* occur if you are diagnosed as having prostate cancer and have an operation for it:**
- **Avoiding URINARY PROBLEMS**
- **Avoiding BOWEL PROBLEMS**
- **Avoiding SEXUAL PROBLEMS**

**We ask you to indicate how important each is to you.**

**In the Annalisa screen you will see on pressing Next, the bars in the middle Values panel should represent the importance you attach to each attribute.**

[
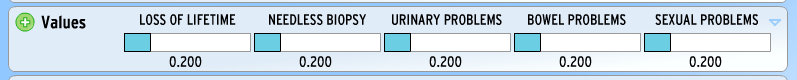
](http://content.screencast.com/users/cafeannalisa/folders/Jing/media/69c724e3-a5fd-4f2f-95e0-c1251c11faa0/weights_panel.png)

**When it opens the 5 are set equally important at 20%.**

**You should now change all the bars until you are happy their lengths represent your actual importance weights for the 5 considerations.**

**Longer means more important, shorter means less important. Make two bars of the same length only if the considerations are equally important.**

**As you move one blue bar, the others adjust themselves, since your weights must add up to 100%. So you will need to play with them for a little while to get what you want.**

**Moving the bars is a matter of dragging the right end with the cursor, as was shown in the video you watched earlier**

**Click 'Next' when you are satisfied with your weighting. (Scroll down if necessary to locate 'Next')**

**What's your result?**

**On the next Annalisa screen you will see the scores for your two options:**

**1. To have a PSA test and the recommended further testing and treatment (if any)**

**2. To not have a PSA test**

**The scores for each option are at the end of the bars in the top panel and the option emerging with the highest score is the longest, darker (orange) bar.**

**The scores are derived from a combination of the importance you have just given to each consideration and the available scientific evidence about the chance of each consideration occurring.**

**Pressing  'Print Screen' on your keyboard will copy the screen to your clipboard and allow you to save it to another document.**

**18** **Would you like to see how each option performs on each of the considerations?**

Please select one item from the list.

- [1] Yes
- [2] No

**In the following Annalisa screen an extra panel appears at the bottom.**

**It contains the Ratings for each of the options on each of the attributes.**

**The longer the bar, the better the option performs.**

**My Decision**

**19** **At this moment, how likely are you to consult your GP within the next 12 months about having a PSA test?**

Please select one item from the list.

- [1] Very Likely
- [2] Likely
- [3] Unlikely
- [4] Very Unlikely
