# Supplementary material for: The Role of Personalised Choice in Decision Support: A Randomized Controlled Trial of an Online Decision Aid for Prostate Cancer Screening
Source: PLoS One. 2016 Apr 6;11(4):e0152999. doi: 10.1371/journal.pone.0152999 (PMC4822955; doi:10.1371/journal.pone.0152999)
Supplement: S8 File — (DOCX) [file pone.0152999.s008.docx]

**Things which may be important to you**

**We think up to ten (10) considerations (or criteria) may be important to you in deciding whether to have a PSA test and, *if required*, further testing and treatment for prostate cancer**

- **Avoiding LOSS OF LIFETIME because of Prostate Cancer**
- **Avoiding LOSS OF HEALTH because of Prostate Cancer**
- **Avoiding a NEEDLESS BIOPSY as a result of a (false) positive PSA test**
- **Avoiding OVERDIAGNOSIS (and needless treatment) as a result of a PSA test detecting a cancer that would not have affected your life or health

  Another three concerns are outcomes *that might* occur if you are diagnosed as having prostate cancer and have an operation for it:**
- **Avoiding URINARY PROBLEMS**
- **Avoiding BOWEL PROBLEMS**
- **Avoiding SEXUAL PROBLEMS

  Three final considerations for you may be**
- **Avoiding the personal BURDEN of TREATMENT for Prostate Cancer**
- **Avoiding creating the BURDEN to CARERS associated with treatment for Prostate Cancer**
- **Avoiding REGRET at not having the test if it later turned out that you had Prostate Cancer that would have benefited from treatment**

**You are the expert!**

**For seven of these considerations there is evidence about how well each option performs and this evidence provides the ratings in the upcoming Annalisa.**

**But for the last three- Treatment Burden, Carer Burden and Regret - you are the expert, so we now ask you to rate them yourself to provide what the Annalisa needs.**

**18** **How great would you find the personal Burden of being treated for diagnosed Prostate Cancer?**

Please select one item from the list.

- [1] Very small
- [2] Small
- [3] Moderate
- [4] Large
- [5] Very large

**19** **How great would you see the Carer Burden resulting from you being treated for diagnosed Prostate Cancer?**

Please select one item from the list.

- [1] Very small
- [2] Small
- [3] Moderate
- [4] Large
- [5] Very large

**20** **How much regret would you anticipate feeling if you did not have a PSA test and were later diagnosed with Prostate Cancer that was affecting your length of life and health?**

Please select one item from the list.

- [1] Little or none
- [2] Some
- [3] A moderate amount
- [4] A great deal
- [5] An enormous amount

**Which considerations do you want to include in your Decision Aid?**

**Use the buttons to indicate which considerations are important enough to you to be included in the upcoming Annalisa. Click the Exclude button for the others.**

|  |  | **Include in my decision aid [1]** | **Exclude [2]** |
| --- | --- | --- | --- |
| **21** | AVOIDING LOSS OF LIFETIME because of Prostate Cancer |  |  |
| **22** | AVOIDING LOSS OF HEALTH because of Prostate Cancer |  |  |
| **23** | AVOIDING a NEEDLESS BIOPSY (and possible consequential treatments) as a result of a false  positive test result |  |  |
| **24** | AVOIDING OVERDIAGNOSIS (and needless treatment) as a result of a PSA test detecting a  cancer that would not have affected your life or health |  |  |
| **25** | AVOIDING moderate to severe URINARY PROBLEMS |  |  |
| **26** | AVOIDING moderate to severe BOWEL PROBLEMS |  |  |
| **27** | AVOIDING moderate to severe SEXUAL PROBLEMS |  |  |
| **28** | AVOIDING the personal BURDEN OF TREATMENT for Prostate Cancer |  |  |
| **29** | AVOIDING creating the BURDEN on CARERS associated with being treated for Prostate Cancer |  |  |
| **30** | Avoiding REGRET at not having the test if it later turned out that you had Prostate Cancer that  would have benefited from treatment |  |  |

**In the Annalisa screen you will see on pressing Next, the bars in the middle Values panel represent the importance you attach to each attribute.**

**The Annalisa screen that you will see will contain only the attributes you chose to include, with equal bar lengths that reflect the number you chose.**

**You should now change all the bar lengths until you are happy they represent your actual importance weights for these considerations.**

**Longer means more important, shorter means less important. Make two bars of the same length only if the considerations are equally important.**

**Moving the bars is a matter of dragging the right end with the cursor, as was shown in the video you watched earlier**

**Click 'Next' when you are satisfied with your weighting. (Scroll down if necessary to locate 'Next')**

**What's your result?**

**On the next Annalisa screen you will see the scores for your two options:**

**1. To have a PSA test and the recommended further testing and treatment (if any)**

**2. To not have a PSA test**

**The scores for each option are at the end of the bars in the top panel and the option emerging with the highest score is the longest, darker (orange) bar.**

**The scores are derived from a combination of the importance you have just given to each consideration and the available scientific evidence about the chance of each consideration occuring.**

**Pressing  'Print Screen' on your keyboard will copy the screen to your clipboard and allow you to save it to another document.**

**31** **Would you like to see how each option performs on each of the considerations?**

Please select one item from the list.

- [1] Yes
- [2] No

**In the following Annalisa screen an extra panel appears at the bottom.**

**It contains the Ratings for each of the options on each of the attributes.**

**The longer the bar, the better the option performs.**

**My Decision**

**32** **At this moment, how likely are you to consult your GP within the next 12 months about having a PSA test?**

Please select one item from the list.

- [1] Very Likely
- [2] Likely
- [3] Unlikely
- [4] Very Unlikely
