# Supplementary material for: The Role of Personalised Choice in Decision Support: A Randomized Controlled Trial of an Online Decision Aid for Prostate Cancer Screening
Source: PLoS One. 2016 Apr 6;11(4):e0152999. doi: 10.1371/journal.pone.0152999 (PMC4822955; doi:10.1371/journal.pone.0152999)
Supplement: S9 File — (DOCX) [file pone.0152999.s009.docx]

**My Decision Quality**

**My Decision Qualit**y **is a tool for YOU to assess - from YOUR perspective - the quality of a decision that has just been taken.**

**We interpret your decision as being the answer you have given to the question:
how likely you think you are to have a PSA test**

**You should recognise the 8 criteria on the next screen as the same ones you met earlier in My Decision Preparation**

**Please now RATE the decision.
In other words, say how well it meets each criterion by clicking on the appropriate button.**

**Please respond to each item in relation to the decision you have just made about PSA testing and the process of making it**

|  |  | **Strongly agree [1]** | **Agree [2]** | **Neither agree/ disagree [3]** | **Disagree [4]** | **Strongly disagree [5]** |
| --- | --- | --- | --- | --- | --- | --- |
| **33** | OPTIONS  **I am clear about the possible OPTIONS for me and what they involve** |  |  |  |  |  |
| **34** | EFFECTS  **I am clear about the possible EFFECTS and outcomes of the options for me** |  |  |  |  |  |
| **35** | IMPORTANCE  **I am clear about the relative IMPORTANCE of the different effects and outcomes for me** |  |  |  |  |  |
| **36** | CHANCES  **I am clear about the CHANCES of the different effects and outcomes happening to me, including the uncertainties surrounding the best estimates** |  |  |  |  |  |
| **37** | TRUST  **I TRUST the information I have been given is the best possible** |  |  |  |  |  |
| **38** | SUPPORT  **I am satisfied with the level of SUPPORT and consideration I received throughout the decision process, especially in regard to communicating at my level** |  |  |  |  |  |
| **39** | CONTROL  **I feel in CONTROL of my decision to the extent I wish** |  |  |  |  |  |
| **40** | COMMITMENT  **I am COMMITTED to acting on my decision** |  |  |  |  |  |

**My Score for Decision Quality**

**On the next screen in the bottom panel you will see the criterion Ratings you just provided.**

**In the middle panel you will find the Weightings for these criteria that you provided in My Decision Preparation**

**In the top panel you will find the Decision Quality Score that combines your Ratings and Weightings.**

**We suggest a score below 50%  is not satisfactory, 50-70% is satisfactory to good, and above 70% very good to excellent.**

**This is your chance to revise your weights if you wish, so  don't press 'Next' until you are fully satisfied that the Weightings in the middle panel are good indicators of the relative importance of the criteria to you.**

**[Reminder: You do this by changing the length of the blue bars in the Weightings panel by placing the cursor on its right end and dragging it to where you want.]**

**41** **Are you interested in finding where the Decision Quality might be improved?**

Please select one item from the list.

- [1] Yes
- [2] No

**Sources of Decision Quality Improvement**

**On the next and final screen you will be able to work out where you might best improve your Decision Quality score if the opportunity exists.**

**The DQ Score bar is now partitioned into 8 segments, representing the contribution of each criterion.**

**If the segment is large this criterion is making a big contribution to your Decision Quality score, if it is small it is making a small contribution.**

**Place the cursor over a segment of the bar and you will see the relevant criterion label below is highlighted.**

**It may be worth looking at some of the small segments, because potentially they could be larger.**

**If you have assigned the criterion a small *Weighting* that's fine.**

**However, if you have assigned it a small *Rating* (with a moderate or large Weighting) then you should explore if and how you might change things for the better. For example, by seeking more information, or more value clarification, or more preferred level of control,  or... whatever the criterion relates to**

**Decision Follow-up**

**42** **Enter here anything you would like to do by way of follow-up to your decision.**

**If you would like to read more about the debate on PSA testing, a chapter written by members of the Sydney School of Public Health appears as soon as you click on 'Submit Responses' at the end of the survey.**

**Thanks for using My Decision Suite for Prostate Cancer Screening.**

**We hope it helped your decision making.**

**That is the end of the aid.**

**Please now give us your feedback and some information**

**Your reactions to the decision aid**

**43** **How surprised are you by the option which emerged with the highest score for you?**

Please select one item from the list.

- [1] not at all
- [2] slightly
- [3] fairly
- [4] very
- [5] extremely

**44** **How difficult was it for you to DECIDE on the weights for the 5 considerations?**

Please select one item from the list.

- [1] Very difficult
- [2] Fairly difficult
- [3] Neither difficult nor easy
- [4] Fairly easy
- [5] Very easy

**45** **How difficult was it for you to ENTER the weights for the 5 considerations?**

Please select one item from the list.

- [1] Very difficult
- [2] Fairly difficult
- [3] Neither difficult nor easy
- [4] Fairly easy
- [5] Very easy

**46** **How well did the contents of the Evidence and Sources sections meet your needs for information?**

Please select one item from the list.

- [1] Very well
- [2-] Well
- [3] Not very well

**47** **Did you print out or capture your Annalisa for PSA testing?**

Yes [1] No [0]

**48** **If you would like to give feedback of any sort, please enter your comments here**

We cannot address medical questions. Please consult your GP.

**A few things we need to know about you to help our research**

**49** **How would you describe your general health?**

Please select one item from the list.

- [1] Excellent
- [2] Very good
- [3] Good
- [4] Fair
- [5] Poor

**50** **How long is it since you last visited your family doctor or another General Practitioner (GP)?**

Please select one item from the list.

- [1] Less than 2 weeks
- [2] 2 weeks to 3 months ago
- [3] 3 to 6 months ago
- [4] 6 to 12 months ago
- [5] 12 months or more
- [6] Never
- [7] Not sure

**51** **Which of the following health covers do you have (excluding Medicare)?**

Please select between 1 and 4 items from the list.

- [1] Private health insurance with extras
- [2] Private health insurance without extras
- [3] Department of Veteran's Affairs white or gold card
- [4] Health care concession card
- [5] none of these
- [6] I don't know

**52** **Where were you born?**

Please select one item from the list.

- [1] Australia
- [2] New Zealand
- [3] UK or Ireland
- [4] Elsewhere in Europe
- [5] Elsewhere in World

**53** **What is your relationship status?**

Please select one item from the list.

- [1] Married
- [2] de facto
- [3] Separated
- [4] Divorced
- [5] Widowed
- [6] Never married

**54** **What is your highest qualification?**

Please select one item from the list.

- [1] Higher degree/Post-graduate diploma/Bachelor degree
- [2] Undergraduate diploma/Associate diploma
- [3] Skilled/Basic vocational qualification
- [4] Has qualification but unsure about the level
- [5] No higher qualifications

**55** **What is your current employment status?**

Please select one item from the list.

- [1] not in the labour force
- [2] employed
- [3] unemployed
- [4] Don't know

**56** **What is your home postcode?**

**You can read more about prostate cancer screening by going to the following University of Sydney website at a time convenient for you:** http://ses.library.usyd.edu.au/bitstream/2123/6835/3/Let-sleeping-dogs-lie.pdf

**Thank you very much for your co-operation. Your responses will be of great help.**

**The MyDecisionSuite (MDS) template is © 2011 Jack Dowie and Mette Kjer Kaltoft and is used with permission**

**MyDecisionQuality(MDQ) instrument (Pre- and Post- elements) is © 2011 Jack Dowie and Mette Kjer Kaltoft and is used with permission**

**MDS: My Prostate Cancer Screening Aid is © 2011 Glenn Salkeld, Michelle Cunich, Jack Dowie, Kirsten Howard**

**Elicia and Annalisa are © 2011 Maldaba Ltd**
