# Supplementary material for: The Role of Personalised Choice in Decision Support: A Randomized Controlled Trial of an Online Decision Aid for Prostate Cancer Screening
Source: PLoS One. 2016 Apr 6;11(4):e0152999. doi: 10.1371/journal.pone.0152999 (PMC4822955; doi:10.1371/journal.pone.0152999)
Supplement: S10 File — (DOCX) [file pone.0152999.s010.docx]

**E-Appendix 1 Personalizing Annalisa**

In order to develop a personalized decision aid for prostate cancer, we identified a set of 10 attributes that we thought would be relevant to decision-making.

Our choice of the five fixed attributes was based primarily on the factors that have been examined in published studies of prostate cancer screening. For example, Andriole et al and Schroder et al have studied the effects of screening on prostate cancer mortality^1,2^ while Sanda et al have examined the effects of treatment for prostate cancer on urinary, bowel and sexual function.^3^

We also based our choice of fixed attributes on the attributes that are included in the majority of existing prostate cancer screening decision aids**^e.g.4-8^** and on the findings of a GP pilot study, examining the usefulness of an earlier version of the Annalisa PSA decision aid,^9^

Through this process, we arrived at a group consensus on our choice of the following five fixed attributes: survival (loss of lifetime), needless biopsy (as a result of a false positive PSA test), and urinary, bowel and sexual functioning problems that could occur if treated for prostate cancer.

Our choice of the five extra’ attributes was also based on the content of existing decision aids, as well as on other elements that have been identified as significant by clinicians, researchers and/or patients.

The “screening pathway” for prostate cancer is a process that includes not only the test itself, and resulting diagnostic and therapeutic procedures, but also more general outcomes for the patient such as **quality of life** (overall health) and the **overall burden of treatment** **^e.g.^** ^3,10,11^—hence our choice of these two attributes as extended attributes

The problem of “**overdiagnosis**” of prostate cancer (the effects of detecting a cancer that would not have otherwise affected quantity or quality of life), is increasingly recognized as a major clinical and public health issue**^e.g.^**^12-14^ and many decision aids make direct and indirect reference to this,**^e.g.^**^4,5,7,8^ hence our decision to include this as one of the extended attributes.

Our decision to include the two “psychosocial” attributes of **burden to carers** and **anticipated regret** was based on the recognition that cancer patients do not have only their own wellbeing in mind—they also worry about being a burden to others^15,16^ —and on the recognition that, when it comes to screening and treatment for cancer, the outcomes that matter are psychological as well as physical—e.g. anticipated regret.^17-19^ The importance of being “safe” rather than “sorry” is also a feature of some existing prostate cancer screening decision aids.**^e.g.^**^4-6^

In this way we reached consensus on the choice of: quality of life, overdiagnosis, burden of treatment, burden to carers, and anticipated regret as our five extended attributes. We acknowledge that it would have been possible to organize the attributes differently in terms of which were fixed and which were extended. It would also have been possible to include other relevant extended attributes (anxiety, depression, occupational function, financial burden etc.) but we felt that many of these would be subsumed under other concepts (e.g. “quality of life”) and we did not want the personalized aid to be unmanageably long.

Once we had agreed upon the five fixed and five extended attributes, we then reviewed the literature to derive the best available evidence for the likelihood of each attribute occurring with and without PSA testing. The probabilities (absolute) of avoiding loss of lifetime, and avoiding needless biopsy were based on a population model of PSA screening by Howard et al 2009 using up-dated data.^20^{Howard, 2{Howard, 2009 #2918}009 #2918} The probabilities of avoiding the possible side effects of treatment for prostate cancer (i.e. urinary incontinence, bowel dysfunction and impotence) and avoiding loss of health (quality of life) were derived from a cohort study by Smith et al,^21^ in which the quality of life of men treated for prostate cancer was compared to a matched cohort of men who had not had prostate cancer. The data from Smith et al were incorporated, with the author’s permission, into the Howard et al model to generate the probabilities of these events. The probability of avoiding overdiagnosis was derived from the ERSPC trial.^2^

Where there were no published data for an attribute (such as burden of treatment, burden to carer and anticipated regret), participants were asked to rate the level of difficulty they would encounter in avoiding each outcome on a 5 point Likert scale (1 = very small and 5 = very large) as part of the online survey that was completed prior to the decision aid.

**
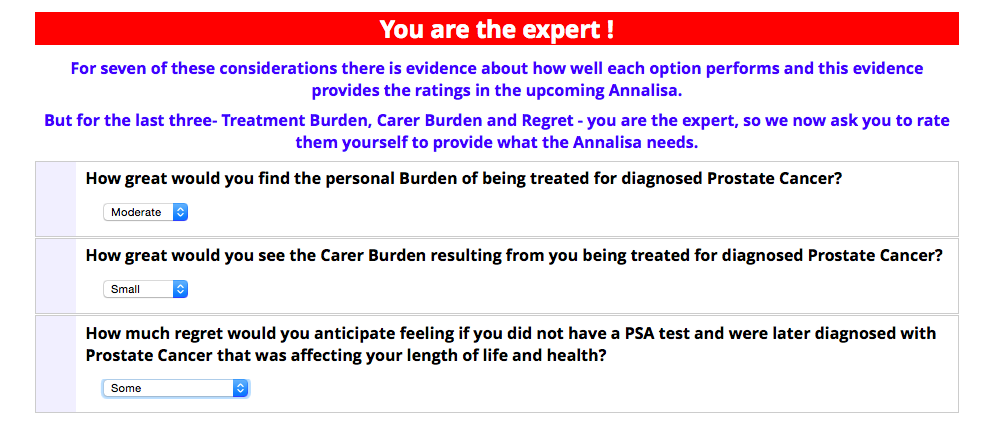
**

All probabilities were refined according to degree of risk of prostate cancer. Low-risk men were those with no first-degree relatives affected by prostate cancer. Men with one affected first-degree relative were considered to be at moderate risk, and men with two or more affected first-degree relatives were considered to be at high risk.^20^

The decision support tool for prostate cancer was then divided into two different versions for the purposes of testing the effects of personalizing decision support. In one version (active comparator), users were not given any choice about the attributes that will be included in the decision support tool—they were forced to consider all five of survival (lifetime), needless biopsy (as a result of a false positive PSA test), and urinary, bowel and sexual functioning problems that could occur if treated for prostate cancer. Those in the personalized choice (active intervention) group were asked to choose as few or as many as they wish of the five “standard” attributes, as well as of the five other “extended attributes”: general health, overdiagnosis, burden of treatment, carer burden, and anticipated regret. All attributes were expressed in the positive frame (the chance of *avoiding* loss of lifetime, needless biopsy, etc.) to ensure consistency of user interpretation.

**1.** Andriole GL, Grubb RL, III, Buys SS, et al. Mortality Results from a Randomized Prostate-Cancer Screening Trial. *N Engl J Med.* Mar 26 2009;360(13):1310-1319.

**2.** Schroeder FH, Hugosson J, Roobol MJ, et al. Screening and Prostate-Cancer Mortality in a Randomized European Study. *N Engl J Med.* Mar 26 2009;360(13):1320-1328.

**3.** Sanda MG, Dunn RL, Michalski J, et al. Quality of life and satisfaction with outcome among prostate-cancer survivors. *N Engl J Med.* Mar 20 2008;358(12):1250-1261.

**4.** Gattellari M, Ward JE. Does evidence-based information about screening for prostate cancer enhance consumer decision-making? A randomised controlled trial. *J Med Screen.* 2003 2003;10(1):27-39.

**5.** National Health Service. PSA (Prostate Specific Antigen) testing for prostate cancer. <http://www.cancerscreening.nhs.uk/prostate/prostate-patient-info-sheet.pdf>.

**6.** Dorfman C, Williams R, Kassan E, et al. The development of a web- and a print-based decision aid for prostate cancer screening. *BMC Med Inform Decis Mak.* 2010;10.

**7.** Urological Society of Australia and New Zealand. The early detection of prostate cancer in general practice: supporting patient choice. 2007; <http://www.usanz.org.au/uploads/65337/ufiles/PDF/6_PSA_decision_card_041007.pdf>.

**8.** American Cancer Society. Testing for prostate cancer. 2010; <http://www.cancer.org/acs/groups/content/@editorial/documents/document/acspc-024618.pdf>.

**9.** Cunich M, Salkeld G, Dowie J, et al. Integrating Evidence and Individual Preferences Using a Web-Based Multi-Criteria Decision Analytic Tool An Application to Prostate Cancer Screening. *Patient.* Sep 1 2011;4(3):153-162.

**10.** Malcolm JB, Fabrizio MD, Barone BB, et al. Quality of Life After Open or Robotic Prostatectomy, Cryoablation or Brachytherapy for Localized Prostate Cancer. *J Urol.* May 2010;183(5):1822-1828.

**11.** Namiki S, Arai Y. Health-related quality of life in men with localized prostate cancer. *Int J Urol.* Feb 2010;17(2):125-138.

**12.** Pashayan N, Duffy SW, Pharoah P, et al. Mean sojourn time, overdiagnosis, and reduction in advanced stage prostate cancer due to screening with PSA: implications of sojourn time on screening. *Br J Cancer.* Mar 31 2009;100(7):1198-1204.

**13.** Bangma CH, Roemeling S, Schroder FH. Overdiagnosis and overtreatment of early detected prostate cancer. *World J Urol.* Mar 2007;25(1):3-9.

**14.** Barry MJ, Mulley AJ, Jr. Why Are a High Overdiagnosis Probability and a Long Lead Time for Prostate Cancer Screening So Important? *J Natl Cancer Inst.* Mar 18 2009;101(6):362-363.

**15.** McPherson CJ, Wilson KG, Murray MA. Feeling like a burden: Exploring the perspectives of patients at the end of life. *Soc Sci Med.* Jan 2007;64(2):417-427.

**16.** Simmons LA. Self-perceived burden in cancer patients - Validation of the self-perceived burden scale. *Cancer Nurs.* Sep-Oct 2007;30(5):405-411.

**17.** Connolly T, Reb J. Regret in cancer-related decisions. *Health Psychol.* Jul 2005;24(4):S29-S34.

**18.** Sandberg T, Conner M. A mere measurement effect for anticipated regret: Impacts on cervical screening attendance. *Br J Soc Psychol.* Jun 2009;48(2):221-236.

**19.** Sorum PC, Mullet E, Shim J, Bonnin-Scaon S, Chasseigne G, Cogneau J. Avoidance of anticipated regret: The ordering of prostate-specific antigen tests. *Med Decis Making.* Mar-Apr 2004;24(2):149-159.

**20.** Howard K, Barratt A, Mann G, Patel M. A Model of Prostate-Specific Antigen screening outcomes for low- to high-risk men: Information to support informed choices. *Arch Int Med.* 2009;169(17):1603-1610.

**21.** Smith D, King M, Egger S, et al. Quality of life three years after diagnosis of localised prostate cancer: population based cohort study. *BMJ.* 2009;339(Nov 27):b4817.
