# Supplementary material for: The Role of Personalised Choice in Decision Support: A Randomized Controlled Trial of an Online Decision Aid for Prostate Cancer Screening
Source: PLoS One. 2016 Apr 6;11(4):e0152999. doi: 10.1371/journal.pone.0152999 (PMC4822955; doi:10.1371/journal.pone.0152999)
Supplement: S11 File — (DOCX) [file pone.0152999.s011.docx]

**E-Appendix 2: MyDecisionQuality explanation and weightings items**

MyDecisionQuality is a web-based instrument that can generate a patient-reported measure of the quality of a particular decision. It is grounded in multi criteria decision analysis. Using a simple expected value algorithm, it calculates a decision quality score based on eight criteria, (listed below), which are rated on a scale of 1 to 5. It can also be used prior to decision-making in order to ascertain which decision quality criteria are most important to participants.^1^

**Version presented to participants before completion of the decision aid**


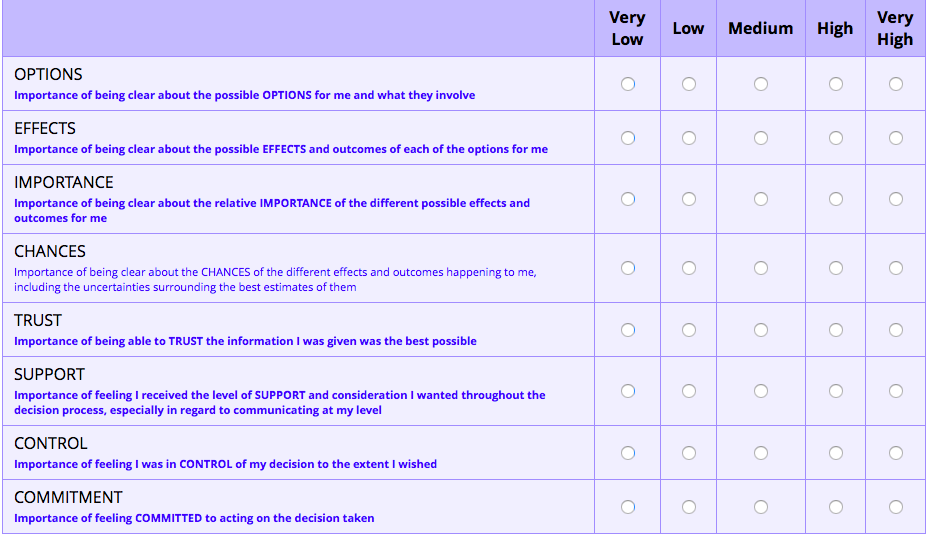


**Version presented to participants after completion of the decision aid**


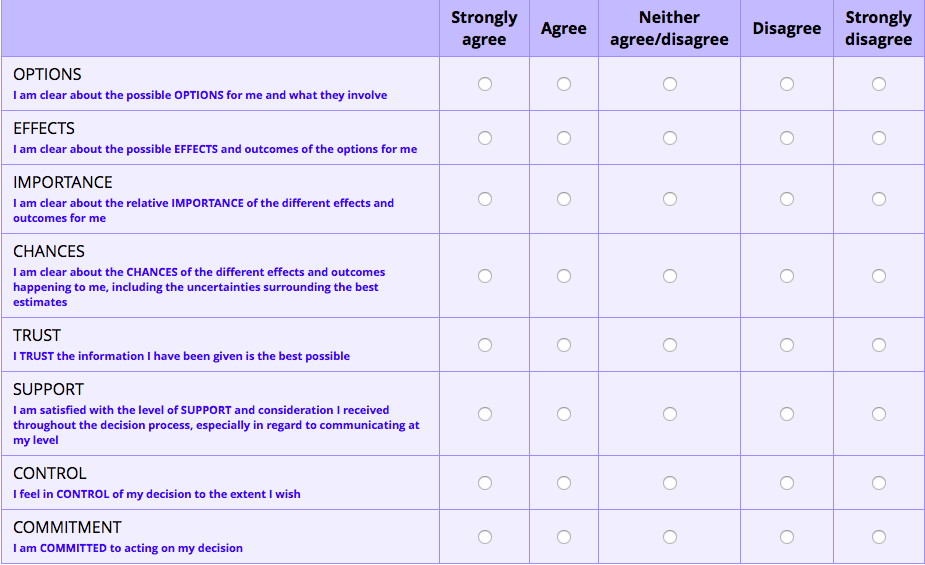


**1.** Kaltoft M, Cunich M, Salkeld G, Dowie J. Assessing decision quality in patient-centred care requires a preference-sensitive measure. *J Health Serv Res Policy.* Apr 2014;19(2):110-117.
